# Contaminants of Emerging Concern in Tomatoes Grown in Sludge-Amended Peat: Uptake, Translocation and Risk Assessment

**DOI:** 10.3390/toxics13121013

**Published:** 2025-11-22

**Authors:** Eirini Andreasidou, Ana Kovačič, Lorena Manzano-Sánchez, David Heath, Marina Pintar, Nina Kacjan Maršič, Urška Blaznik, Amadeo Rodríguez Fernández-Alba, Maria Dolores Hernando, Ester Heath

**Affiliations:** 1 Department of Environmental Sciences, Jožef Stefan Institute, Jamova Cesta 39, 1000 Ljubljana, Slovenia; 2 International Postgraduate School Jožef Stefan, Jamova Cesta 39, 1000 Ljubljana, Slovenia; 3 European Union Reference Laboratory for Pesticide Residues in Fruits and Vegetables, Department of Chemistry and Physics, Agrifood Campus of International Excellence (ceiA3), University of Almería, La Cañada de San Urbano, 04120 Almería, Spain; 4 Department of Agronomy, Biotechnical Faculty, University of Ljubljana, Jamnikarjeva 101, 1000 Ljubljana, Slovenia; 5 Environmental Health Centre, National Institute of Public Health, Trubarjeva 2, 1000 Ljubljana, Slovenia; 6 Department of Desertification and Geo-Ecology, Experimental Station of Arid Zones, Consejo Superior de Investigaciones Científicas, La Cañada de San Urbano, 04120 Almería, Spain

**Keywords:** *Solanum lycopersicum* L., bioaccumulation, pharmaceuticals, personal care products, bisphenols, pesticides, hormones, LC-MS/MS, dietary exposure, ecological risk assessment

## Abstract

Although sewage sludge in agriculture can promote circular economy goals, concerns remain about the transfer of contaminants of emerging concern (CECs) into crops and soils. This study evaluated the uptake and risk of 27 CECs in tomatoes cultivated in peat substrate amended with stabilised anaerobically digested (dried) sludge from a local municipal wastewater treatment plant at two rates corresponding to nitrogen and nitrogen/potassium requirements. Peat substrate served as the control. Additional treatments included CEC-spiked media and peat amended with non-dried sludge. Analysis was performed with LC–MS/MS. In tomato fruits, ibuprofen (15.8 ng/g) and triclosan (17.9 ng/g) were quantified at the low amendment rate, while caffeine (381 ng/g), carbamazepine (18.1 ng/g), ciprofloxacin (306 ng/g) and ibuprofen (5.3 ng/g) were quantified at the high amendment rate. Dietary exposure estimates were below the health-based reference values for most compounds; however, a potential risk was identified for bisphenol S when non-dried anaerobically digested sludge was applied. Soil risk quotients (RQ > 1) for several CECs at the end of the experiment indicate possible ecological concern. These findings emphasise that monitoring CECs in sludge-amended soil remains essential to ensure the safety of sludge reuse in agriculture.

## 1. Introduction

The sustainable management of stabilised sewage sludge (also referred to as dewatered biosolids), which is a by-product of wastewater treatment, remains challenging due to contaminants, compositional variability, and regulatory constraints on its reuse [1]. Despite this, sludge is a source of nitrogen, phosphorus, organic carbon, and micronutrients, and its application in soil can improve both chemical and physical properties, including cation exchange capacity, field capacity, and bulk density [2]. Early studies have shown that the application of sludge increased crop yields by an average of 7% [3]. In Europe, approximately 35–50% of total sludge management involves agricultural use [4]. Its use in agriculture also aligns with the EU’s Circular Economy Action Plan, which promotes resource recovery and waste valorisation [5]. Compared to landfilling and incineration, it offers a cost-effective way of recycling nutrients while mitigating the environmental impact of waste sludge disposal [5].

At the European level, the agricultural use of treated sewage sludge is regulated under Council Directive 86/278/EEC, which sets limits for heavy metals to protect the health of soil, plants, animals, and humans [6]. National legislation across EU Member States often imposes different requirements or further limitations, reflecting both environmental concerns and varying levels of sludge reuse in agriculture [7,8], with most countries not supporting the use of sludge on crops intended for direct human consumption due to the risks posed by heavy metals and pathogens [9]. However, many believe that the reuse of sludge should be reconsidered, given the increasing volume of sludge produced, rising disposal costs (e.g., incineration), and the push toward sustainability.

While heavy metals and pathogens are already considered, risks from contaminants of emerging concern (CECs) remain unaddressed [10,11,12]. These include pharmaceuticals, personal care products, pesticides [13], and industrial chemicals [14] that are not yet regulated. Their persistence through wastewater treatment and bioactivity raises concerns about their potential accumulation in amended soils, subsequent transfer into crops, and entry into the food chain, where they can pose a risk to human health [15].

Carbamazepine, for example, has been shown to be taken up by different vegetables, including carrot, celery, lettuce, tomato, pak choi and wheat grown in biosolid-amended soils [16,17]. Similarly, trace amounts of triclocarban and triclosan have been detected in various plant parts [13]. Studies also indicate that the uptake and translocation of CEC depend not only on their physicochemical properties (e.g., log Kow, sorption affinity) but also on soil characteristics, e.g., pH, CaCO_3_ content, organic matter and number of basic cation exchangeable sites [15,18], as well as sludge properties including pH, total organic carbon, total suspended solids and heavy metals content [19,20].

Fewer studies, however, have examined the translocation of CECs in tomatoes grown in amended soils (Table 1) [21,22]. Among them, four looked at uptake and translocation and found the presence of CECs, including carbamazepine and its metabolites in leaves and fruits [17], triclocarban present in root > shoot > fruit [22] and Per- and Polyfluoroalkyl Substances (PFASs), with higher translocation of short-chain ones, to aerial tissues [23,24]. Knowledge gaps also persist regarding the risks associated with agricultural applications. For instance, studies typically focus on a smaller group of compounds, such as triclosan, triclocarban, and carbamazepine, rather than on realistic CEC mixtures present in sludge [25,26].

The present study examines the uptake and translocation of 27 CECs, including selected pesticides (2), pharmaceuticals (9), personal care product ingredients (6), hormones (5), stimulants (1) and industrial chemicals (4), in tomato plants cultivated in pots containing peat substrate amended with dried anaerobically digested sludge, at two amendment levels covering (1) nitrogen and (2) nitrogen and potassium plant requirements. In addition, the growth media were spiked with CECs at levels higher than typically found in sewage sludge to enhance the likelihood of detecting CECs in tomato tissues. In addition, peat substrate was amended with non-dried sludge. The study aimed to evaluate the impact of sludge amendment on fruit yield and the presence of CECs in plant tissues and growing media (soil risk quotients), as well as to assess dietary exposure from consuming tomatoes (*Solanum lycopersicum* L. cv. Rally) grown in sludge-amended media.

## 2. Materials and Methods

### 2.1. Reagents and Chemicals

Analytical-grade standards (>97%) were utilised, including acetamiprid, azithromycin, benzophenone, bisphenol F (BPF), bisphenol AF (BPAF), bisphenol S (BPS), caffeine, carbamazepine, ciprofloxacin, clarithromycin, diclofenac sodium salt, dimethomorph, erythromycin, estrone (E1), 17α-ethynylestradiol (EE2), ibuprofen, methylparaben, naproxen, progesterone, propylparaben, testosterone, tonalide, triclocarban, and triclosan. These were purchased from Sigma-Aldrich (Steinheim, Germany), bisphenol A (BPA) was obtained from Merck (Darmstadt, Germany); amoxicillin trihydrate, acquired from Santa Cruz Biotechnology (Santa Cruz, CA, USA); and beta-estradiol (E2), purchased from Thermo Fisher Scientific (Waltham, MA, USA). Detailed compound information is provided in Supplementary Table S1. A selection of isotopically labelled internal standards was used, including BPA-d_16_, ^13^C_3_-caffeine, carbamazepine-d_10_, clothianidin-d_3_, ^13^C_6_-diclofenac, ibuprofen-d_3_, ^13^C_6_-methylparaben, and ^13^C_6_-propylparaben, all purchased from Sigma-Aldrich (Steinheim, Germany). Additional labelled compounds included oxybenzone-d_5_ from C/D/N Isotopes Inc. (Pointe-Claire, QC, Canada), ^13^C_12_-triclosan from Cambridge Isotope Laboratories (Tewksbury, MA, USA), ^13^C_2_-beta-estradiol from Campro Scientific (Berlin, Germany), and both ^13^C_12_-BPS and ^13^C_12_-BPF from CanSyn Chem. Corp. (Toronto, ON, Canada).

Methanol (MeOH), acetonitrile (MeCN), and water, all of LC-MS grade, were obtained from J.T. Baker (Deventer, The Netherlands). Ultrapure water (18.0 MΩ·cm at 25 °C) was produced using a Milli-Q water purification system (Merck Millipore, Darmstadt, Germany). Formic acid (FA) and ammonium fluoride (NH_4_F) were purchased from Sigma-Aldrich (St. Louis, MI, USA), and ethylenediaminetetraacetic acid disodium salt dihydrate (Na_2_EDTA) was supplied by Kemika d.d. (Zagreb, Croatia). Individual stock solutions of each analyte were prepared in MeOH at 1 mg/mL. From stock solutions, working solutions (10, 100, and 1000 ng/mL) were prepared through serial dilutions in MeOH. The internal standard mixture (1 µg/mL) was similarly prepared in MeOH by combining isotopically labelled solutions of individual compounds (1 or 0.1 mg/mL). All prepared solutions were stored in amber glass vials at 4 °C.

### 2.2. Experimental Design and Treatments

Pot experiments were conducted during the 2022 growing season under glass at the University of Ljubljana (Slovenia). Tomato seedlings (*Solanum lycopersicum* L., cv. Rally) were first cultivated for a period of eight weeks, until each plant reached the 5–6 leaf developmental stage, before transplanting to the experimental pots. Each pot was filled with 1.7 kg of peat substrate (Huminsubstrat N8, Neuhaus, Germany; a high-quality white peat mixture with frozen black peat, clay and humic acids) with and without sludge, according to the specific treatment (Figure S1). The characteristics of the peat substrate are provided in Table S2.

Stabilised sludge was obtained from a local wastewater treatment plant (Domžale-Kamnik WWTP, Domžale, Slovenia). The WWTP treats approximately 25,000 m^3^/day of wastewater, with 8% from industrial sources and 92% from municipal and rainwater sources. It is designed to handle an organic load equivalent to 125,000 population equivalents (PE). The dry matter content of the anaerobically digested sludge was 32%. For dried sludge application, the sludge was dried (up to 200 °C), crushed and sieved (nylon sieve, size 2 mm) (Figure S2) as a basic pretreatment, since in real case application it undergoes treatment to facilitate easier storage, transportation and application, as well as reducing biological hazards [30]. After the experiment, the peat substrate was handled and disposed of in accordance with Slovenian waste management legislation [31].

The nutrient content of the dried sludge is given in Table S3 [32,33,34,35,36]. Specifically, the dried sludge contained 35,300 mg/kg of N (mostly as NH_4_^+^), with 30% of the N potentially transformable from NH_4_^+^ to NO_3_^−^; 2238 mg/kg of P_2_O_5_; and 1000 mg/kg of K_2_O. The experiment included sludge-based, CEC-spiked, and control treatments (Table 2). For the control, only peat substrate (PC) was used. For sludge amendments, two application rates were tested: SP1—dried sludge mixed with peat substrate (45 g per pot, 320 mg N/kg of peat substrate) to meet nitrogen requirements and SP2—to meet N and K requirements (545 g per pot, 330 mg K/kg of peat substrate) for tomato cultivation [37].

The same treatments were then spiked with CECs, resulting in concentrations of individual CEC of SP3: 0.3 mg/kg and SP4: 3.5 mg/kg. Spiking was achieved by adding an ethanol solution of CECs to the dried sludge (SP3 and SP4) or peat (P1 and P2), which were left overnight at room temperature to allow the ethanol to evaporate. The resulting mixture was then homogenised into the peat substrate in the test pots. Finally, treatment with anaerobically digested sludge without an additional drying step (SP5), consisting of a 1:1 (*w*/*w*) mixture, was applied to evaluate CEC uptake from the sludge without any additional treatment.

The pots were placed at a plant density of 3.2 plants/m^2^. All plants were irrigated with potable water as needed, based on the reference evapotranspiration of 510 mm recorded at the Ljubljana Bezigrad weather station for the duration of the experiment (Figure S3) [38]. Fertiliser (ENTEC^®^ mineral fertiliser; NPK 14:7:17; Eurochem, Mannheim, Germany) was applied only to treatments without sludge (PC, P1, P2), covering nitrogen (320 kg/ha, corresponding to 160 mg N/kg soil), phosphorus (160 kg/ha P_2_O_5_, corresponding to 80 mg P/kg soil), and potassium (K_2_O: 330 kg/ha, corresponding to 165 mg K/kg soil) as part of the standard growing procedure.

For pots amended with sludge (SP1–5), deficiencies in plant growth/health were observed at 56 days after planting, including dried and yellow leaves (Figure S4). As a result, fertiliser was added to two of the five replicates to evaluate its effect, since reduced growth was attributed to nutrient deficiency. Foliar addition of Ca and B began 61 days after planting and was applied a total of three times to prevent blackening (Blossom End Rot) of the tomato fruits due to a calcium deficiency.

### 2.3. Sample Collection

Tomato fruits were hand-harvested at the fully ripe stage. The first harvest was performed 49 days after planting, and was followed by ten additional harvests, distributed across three defined sampling periods. The final sampling took place at 117 days after planting. During each sampling, mature fruits were placed into plastic bags, transferred to the laboratory, washed, weighed to evaluate yield and stored whole at −20 °C. Prior to sample preparation, frozen fruits were cut into pieces, weighed, freeze-dried and ground. Composite samples were prepared based on treatments, either by pooling fruits from each plant or by sampling period.

At the end of the experiment, plant tissues (roots, stems, and leaves) were collected and air-dried. Each plant part was ground using a stainless steel-bladed mill and then passed through a 2000 µm mesh sieve. In parallel, peat substrate samples were collected in triplicate from the pots at planting (day 0) and at day 117.

### 2.4. Sample Preparation

Sample preparation protocols for the analysis of contaminants of emerging concern (CECs) in tomato fruits, plant tissues (leaves, roots, and stems), and soil were followed using previously validated protocols [39]. Briefly, for tomato fruits, freeze-dried composite samples were spiked with isotope-labelled internal standards, extracted using solid–liquid extraction with MeOH containing 0.1% FA, followed by solid-phase extraction (SPE) using Oasis Prime HLB cartridges. Final eluates were dried under N_2_, reconstituted in MeOH:Milli-Q (1:1), filtered, and stored at −20 °C prior to LC-MS/MS analysis. Plant part samples (1 g dry weight) were extracted using QuEChERS with acidified MeCN (1% FA), followed by automated µSPE (PAL^®^ RTC autosampler). Extracts were eluted, transferred into LC vials, and stored at −20 °C until analysis. Peat samples were extracted similarly to soil (0.5 g compared to 1 g used in the original method) using solid–liquid extraction with MeOH and 0.1% FA (3 × 4 mL). The solvent from the combined extracts was removed under N_2_, and the residue reconstituted in MeOH:Milli-Q (1:1). The mixture was then filtered and stored at −20 °C.

To determine CECs in dried sludge, 0.5 g of the sample was weighed into a 15 mL centrifuge tube and spiked with 25 µL of isotope-labelled internal standard mixture (1 µg/mL). Acetonitrile (1 mL) was added, and the sample was vortexed for 30 s and shaken overnight (200 rpm). Then, 4 mL MeOH with 0.1% FA was added, followed by 10 min sonication and centrifugation at 9000 rpm for 10 min. The supernatant was transferred to a 250 mL vial, and the residue was re-extracted twice. The combined extracts were diluted with 90 mL of Milli-Q water and loaded onto Oasis Prime HLB cartridges (500 mg, 6 mL). After washing with 10% MeOH in MQ (10 mL) and drying under vacuum for 30 min, analytes were eluted with 6 mL MeOH:MeCN (1:1) with 0.1% FA in three 2 mL aliquots. Eluates were dried under N_2_ at 40 °C, reconstituted in 0.4 mL MeOH:Milli-Q (1:1), filtered, and stored at −20 °C before analysis.

### 2.5. LC-MS/MS Analysis

Samples were analysed using a liquid chromatograph (UHPLC; Shimadzu Nexera X2, Kyoto, Japan) coupled to a triple quadrupole-linear ion trap mass spectrometer (4500 QTRAP, Sciex, Framingham, MA, USA). Chromatographic separation was performed at ambient temperature using a Poroshell 120 EC-C18 column (3.0 × 100 mm, 2.7 µm; Agilent Technologies, Santa Clara, CA, USA). Electrospray ionisation (ESI) was applied in both positive and negative modes. For the positive mode, the mobile phases consisted of acetonitrile (A) and 0.1% FA in water (B). For the negative mode, 0.1% NH_4_F in methanol (A) and water (B) was used. Analytical parameters are described in detail in [40].

The method was validated according to DG SANTE guidelines, assessing linearity, accuracy, recovery, matrix effects, LOD, and LOQ [41,42]. Validation results for tomato and plant parts are provided elsewhere [39]. For tomatoes, LOQs ranged between 1.11 and 287 ng/g, while for plant parts, the range was between 25 and 100 ng/g. For the peat substrate, linearity was evaluated using the calibration curves, resulting in R^2^ values ranging from 0.978 to 0.999, while LODs and LOQs were between 0.04 and 11.1 ng/g and 0.13–36.4 ng/g, respectively. Recoveries ranged from 55% to 160%, with most values close to 100%, and RSDs remained below 20%. For sludge, R^2^ values ranged from 0.902 to 0.999, LODs and LOQs were between 0.04 and 1.940 ng/g and 0.15–6.402 ng/g, respectively. Recoveries ranged from 41% to 157%, with most values close to 90%, while the RSD remained below 20%. Detailed results and clarifications on validation criteria are provided in the Supplementary Information (SI-2.2, Table S4).

### 2.6. Bioconcentration, Root Concentration and Translocation Factors

The bioconcentration factor (BCF), root concentration factor (RCF) and translocation factor (TF) were calculated to assess the uptake and internal distribution of CECs in tomato plants. Concentrations of CECs are based on dry weight (d.w.). The BCF was estimated as the ratio of the concentration of each compound in tomato fruit to the corresponding concentration in the peat substrate at the start of the experiment (day 1), as shown in Equation (1).

(1) $$BCF=\frac{Cf}{Cp}$$

Here, *Cf* and *Cp* are the concentration of CEC (μg/kg d.w.) in fruits and in peat substrate. The same approach was applied to estimate the RCF of tomato plants based on Equation (2):

(2) $$RCF\;=\frac{Cr}{Cp}$$

where *Cr* is the concentration of CEC in roots (μg/kg d.w.). Similarly, TF was calculated to assess compound translocation from roots to aerial plant parts, including leaves, stems, and fruits, in various combinations such as stem/root, leaf/root, and fruit/root based on Equation (3):

(3) $$TF\;=\frac{Ca}{Cr}$$

where *Ca* is the concentration of CEC (μg/kg d.w.) in individual aerial plant parts, including stems, leaves and fruits. A TF < 1 indicates that most of the compound remains in roots, whereas a TF > 1 suggests the translocation and potential accumulation in the rest of the tissues [43].

### 2.7. Human Health Risk Assessment

Dietary exposure was estimated for children (toddlers), adults and adolescents by combining data from composite samples (transformed to fresh weight, considering that the evaluated tomato fruits contained 95% water) with the corresponding consumption levels from the EFSA Comprehensive European Food Consumption Database [44]. The consumption data were selected based on the Exposure hierarchy up to Level 7 (Vegetables and vegetable products up to Tomatoes) for the Slovenian Population (Table S5). Default body weight values suggested by EFSA were used for further calculations. Chronic dietary exposure from fresh tomato consumption was calculated for all CEC groups of chemicals, based on both average and high consumption levels (the latter representing the 97.5th percentile of the distribution) [45]. The estimated daily intake (EDI), expressed in mg/kg body weight (bw) per day, was calculated using Equation (4):

(4) $$EDI=\frac{Cff\;\times FC\;}{bw}$$

Here, EDI is given as mg/kg bw/day, *Cff* is the concentration of individual CEC (mg/kg fresh weight) in tomato fruit, FC is the food consumption rate (kg/day) for tomatoes in Slovenia, and bw is the corresponding body weight (kg) for the selected population group. For pesticide residues, an acute exposure was calculated using the International Estimate of Short-term Intake (IESTI) model [46]. This calculation combines the consumed portion of the food product eaten within one meal or day, reflecting the upper tail (the 97.5th percentile) of the distribution of food consumption data (Table S5), with the highest residue level in this food product. The IESTI, expressed in mg/kg body weight (bw) per day, was calculated using Equation (5):

(5) $$IESTI=LPup\;\times \frac{HRup\;}{bw}$$

where IESTI is given as mg/kg bw/day, LPup is the acute large portion for unprocessed tomatoes in Slovenia, HRup is the highest measured concentration of pesticide residue (mg/kg fresh weight) in tomato fruit, and bw is the corresponding body weight (kg) for the selected population group. The results of chronic and acute dietary exposure for each compound were compared with health-based guidance values (HBGV), including Acceptable Daily Intake (ADI), Acute Reference Dose (ARfD), Tolerable Daily Intake (TDI) or No Observed Adverse Effect Level (NOAEL) values from the literature (Table S6) [47,48,49,50,51,52,53,54,55], by calculating the hazard quotient (HQ). HQ was calculated from the ratio of estimated dietary intake (EDI) to health-based guidance values (HBGV), following Equation (6):

(6) $$HQ=\frac{EDI\;}{HBGV}$$

A hazard quotient > 1 indicates a higher daily intake than the HBGV, suggesting a potential risk of exposure [56]. It should be noted that the current assessment refers only to the risk associated with the consumption of tomatoes grown in sludge-amended peat substrate, as a single exposure pathway. Thus, the total exposure from other potential sources is not taken into account.

### 2.8. Ecological Risk Assessment

To evaluate the ecological risk posed by CECs in amended peat substrate, measured environmental concentrations (MECs) from different sampling periods were compared to predicted no-effect concentrations (PNEC). This assessment followed the guidance of the European Chemicals Agency (ECHA) [57].

In the absence of terrestrial ecotoxicological data for the compounds investigated, soil PNEC values were estimated using the equilibrium partitioning method (EPM), based on aquatic toxicity data. PNEC values for freshwater organisms (µg/L) were obtained from the Norman Ecotoxicology Database, which includes experimental data or QSAR predictions suitable for preliminary screening and prioritisation [58]. Soil PNEC values (PNECsoil, µg/kg) were calculated by multiplying the aquatic PNEC values (PNECwater, µg/L) by the soil–water distribution coefficient (Koc, L/kg) of each compound, based on Equation (7) and provided in Table S7.

(7) $$PNECsoil=\left(0.1176+0.01764\times Koc\right)\times PNECwater$$

Equation (7) assumes equilibrium between soil and pore water and includes standard parameters for soil bulk density and porosity [59,60]. This approach is suitable for substances with log Kow values between 3 and 6 and is commonly applied as a screening process for risk when terrestrial toxicity data are unavailable. Although the EPM may either overestimate or underestimate actual risk to soil organisms, it is accepted as a conservative proxy [61].

Risk quotients (RQs) were calculated for each compound as the ratio of MEC to PNEC_soil_, according to Equation (8):

(8) $$RQ=\;\frac{MEC\;}{PNECsoil\;}$$

Interpretation of RQ values followed standard thresholds: RQ < 0.1 indicates low risk, 0.1 ≤ RQ < 1 denotes medium risk, and RQ ≥ 1 signifies a potential high ecological risk [62].

### 2.9. Data Processing and Analysis

Processing of data, statistical analysis and visualisation for all measurements concerning the concentrations of CEC in different samples were carried out using R (version 4.3.3) [63]. For each matrix, concentrations < LOQ were imputed by random draws from a uniform distribution between 0 and LOQ, using matrix-specific LOQs, to preserve variability. To reduce skewness and meet the assumptions of statistical tests, concentrations were log-transformed to the base 10. Normality was tested using the Shapiro–Wilk test; the log_10_-transformed data met the assumption of normal distribution. For tomato data, pairwise comparisons of treatments were performed within each treatment and per compound using Welch’s two-sample *t*-tests, with effect sizes estimated by Cohen’s d. A result was considered statistically significant when *p* ≤ 0.05 and |d| > 1.

Temporal changes in the peat substrate dataset were tested per compound and treatment using an independent two-sample Welch’s *t*-test, which does not assume equal variances between groups, making it more robust for environmental data. A difference in concentration was considered significant if *p* ≤ 0.05 and the effect size |d| > 1. For tomato yield data, which were not normally distributed, a Kruskal–Wallis test was performed across treatments, followed by pairwise Wilcoxon rank-sum tests for (i) all unique treatment pairs and (ii) fertilised vs. non-fertilised within each treatment (*p* ≤ 0.05). Finally, to explore the relationship between fruit number and total yield, a non-parametric Spearman’s rank correlation analysis was conducted.

## 3. Results and Discussion

The levels of CECs were evaluated in peat substrate, sludge and plant parts (fruits, leaves, stems, roots) across the different treatments. For all samples, concentrations are reported as dry weight (d.w.) (Supplementary Information, SI-3).

### 3.1. CEC in Sludge and Amended Peat Substrate

Prior to amendment, dried sludge was analysed separately before mixing (Table S8), and samples from all pots were collected before planting (Table S9). The control pots (PC) showed very low background contamination, with only two compounds (BPS and Naproxen) being quantified with values close to their LOQs. Regarding spiked peat substrate treatments (P1, P2), although the nominal concentrations after spiking were 300 and 3500 ng/g, respectively, the average CEC concentration for P1 was 296 ± 136 ng/g and 2580 ± 1340 ng/g for P2. Methylparaben and propylparaben were not detected in P1, which could be attributed to their rapid degradability under biotic conditions and adsorption to the surfaces of the PVC pots [64,65]. In spiked sludge treatments, concentrations were also close to the nominal spiking levels (SP3: 308 ± 136 ng/g and SP4: 2030 ± 1290 ng/g) for most of the compounds. Variations in concentrations of individual compounds may result from different processes, including adsorption to Petri dishes during sludge spiking and poor homogenisation despite thorough mixing, and subsampling a small amount from the pot for analysis. For SP5 (sludge added directly in a 1:1 ratio), 12 compounds were quantified within a range of 1 to 1630 ng/g.

For each treatment, samples were collected at the end of the experiment (day 117) and CEC concentrations were compared with those at the beginning (day 0), to identify any reduction in concentration during the experimental period (Tables S10–S12, Figures S5–S8). For P1, all compounds quantified at the beginning decreased significantly by the end of the experiment, except for tonalide. This musk has been shown to absorb into soil [66], resulting in slow dissipation and its presence in soil for up to one year. In the case of triclocarban, it is an antimicrobial hydrophobic compound, which may explain its recalcitrance [67,68]. For P2, although the spiked CECs were still present, all compounds quantified at the beginning showed a significant decrease by day 117. In the case of SP3 and SP4, all compounds decreased significantly, except for triclocarban.

The observed differences can be explained based on the compounds’ physicochemical properties [69]. The primary parameters are the organic carbon sorption coefficient (Koc) and octanol–water partition coefficients (log Kow), which can indicate the potential for sorption; for example, compounds with log Kow > 3 (tonalide) tend to sorb to the substrate [70]. Sorption involves various mechanisms, including hydrogen bonding with organic matter, van der Waals interactions, charge transfer, cation exchange, and surface adsorption to soil minerals, which can cause the formation of non-extractable residues, limit detection, and reduce bioavailability [69,71]. The studied hormones: 17α-ethynylestradiol, beta-estradiol, estrone, progesterone, and testosterone, showed a significant reduction in concentration (Table S12) in both spiked sludge treatments. This reduction can be attributed to sorption processes, which are correlated with organic matter and moisture content [72]. Moreover, it has been shown that the presence of other hydrophobic compounds may compete with hormones for available binding sites on the substrate [70].

Other factors that can affect the behaviour of CEC in amended soils include the presence of antimicrobial compounds (e.g., triclocarban), which have been shown to alter the soil microbial community and can further influence the persistence of other compounds [71,73]. Aeration can also affect degradation, which is faster in aerobic compared to anaerobic soil [74].

### 3.2. Uptake and Translocation of CECs in Tomato Plant Parts (Roots, Stems and Leaves)

**Figure 1:**
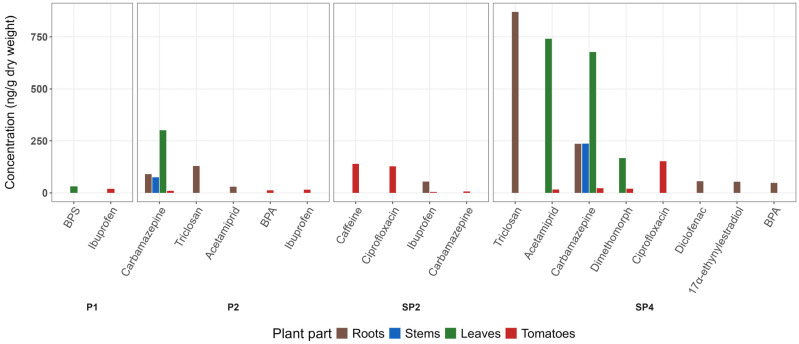
Bar plots of average CEC concentration (ng/g d.w.) in tomato plants grown in different treatments. P1: Peat substrate + CEC (0.3 mg/kg); P2: Peat substrate + CEC (3.5 mg/kg); SP2: Peat substrate + 545 g of dried sludge; SP4: Peat substrate + 545 g of dried sludge + CEC (3.5 mg/kg).

The concentration of CEC in plant parts (roots, stems, leaves) is presented in Table S13. The RCF and TF were calculated for those compounds where values from two different plant parts were available (Table S14). Considering that uptake in plants is concentration-dependent, most compounds were quantified across the spiked treatments (P2 and SP4). At the same time, in a non-spiked amended peat substrate, measurable concentrations were determined in roots and fruits (Figure 1). These patterns are further examined in Section 3.2.1 Roots, Section 3.2.2 Leaves, Section 3.2.3 Stems and Section 3.3 Tomato fruit, where each plant compartment is discussed separately.

#### 3.2.1. Roots

No compounds were detected in the roots of the PC. In samples grown in sludge-amended peat, no compounds were detected in SP1 (low) or SP5 (non-dried sludge), while ibuprofen was quantified (54.4 ng/g) in SP2 (high). Regarding spiked treatments, in P1, no compounds were detected above LOQ. In P2, acetamiprid (29.0 ng/g), carbamazepine (90.3 ng/g), and triclosan (129 ng/g) were >LOQ. Similarly, for SP3, no compounds were quantified, while in SP4, five CECs, 17α-ethynylestradiol (53.5 ng/g), BPA (47.3 ng/g), carbamazepine (235 ng/g), diclofenac (55.5 ng/g), and triclosan (869 ng/g) were quantifiable.

In all treatments, RCF were <1 (Table S14), indicating that the concentration in the roots was lower than that in the peat substrate. The highest RCF was 0.62 for ibuprofen in SP2 treatment. In the case of spiking with CECs at high concentration (P2 and SP4), RCFs were higher in SP4 for carbamazepine (P2: 0.056 and SP4: 0.18) and triclosan (P2: 0.035 and SP4: 0.26) indicating their higher bioavailability in spiked sludge amendment compared to spiked peat substrate, possibly due to the altered physicochemical properties of the substrate (Table S11), such as higher pH values (pH 6.4) and lower C:N ratios (20) in SP4 compared to P2 (pH 5.9 and C:N 40). Also, the addition of sludge can inhibit the degradation of both compounds [75].

Compound-specific physicochemical properties and soil characteristics influence the uptake of CECs by plant roots. Additionally, the concentration of CECs in contaminated soil influences the extent to which CECs are absorbed by plants [76,77]. The pH of the media is also important, as it can affect their uptake and translocation, influenced by both dissociation and hydrophobicity [78].

#### 3.2.2. Leaves

No compounds were identified across the four different non-spiked treatments: PC, SP1, SP2 and SP5. Regarding P1, BPS was quantified in leaves in P1. In P2, only carbamazepine was quantified (301 ng/g). Regarding the spiked sludge amendments (SP3 and SP4), only acetamiprid (740 ng/g), carbamazepine (678 ng/g) and dimethomorph (168 ng/g) were quantified in SP4. The comparison between P2 and SP4, both spiked with the highest levels of CECs, showed that the total concentration in leaves was higher when CECs were derived from the addition of dried sludge (SP4) compared to peat.

#### 3.2.3. Stems

Similar to leaves, no compounds were >LOQ in all treatments except those where a high concentration of CEC was applied (P2 and SP4). In those treatments, carbamazepine was quantified with an average concentration of 74.6 and 236 ng/g, respectively.

#### 3.2.4. Translocation Factors

In terms of translocation from roots to stems and leaves, different compounds were determined; however, TF could be calculated only for carbamazepine in P2 and SP4, as it was the only compound detected across all the different plant parts. Specifically, in P2, the TF of leaves/roots was 3.34, while for stem/roots, 0.83 and 0.12 for fruit/roots, indicating that carbamazepine preferentially translocated from roots to leaves rather than stems or fruits. The same observation also applied to spiked sludge treatment (SP4), where the TFs from roots to leaves, stems, and fruits were 2.64, 0.73 and 0.10, respectively.

Roots can absorb neutral or cationic compounds, since they can cross cell membranes, move with the transpiration stream and accumulate in highly transpiring tissues such as leaves [79]. In peat substrate pH (5.5–6.5), compounds such as acetamiprid, carbamazepine and dimethomorph remain in their neutral form, which can in part explain the findings in leaves and stems. Molecular weight can further influence cell membrane permeability, allowing compounds with a molecular weight < 450 g/mol to transport through the xylem [80]. Nevertheless, CECs in anionic form are often repelled by negatively charged plant cell membranes, thus limiting the movement through tissues, so compounds such as 17α-ethynylestradiol, diclofenac, ibuprofen and triclosan tend to accumulate mainly in roots [81,82].

### 3.3. Bioaccumulation of CECs in Tomato Fruit

**Figure 2:**
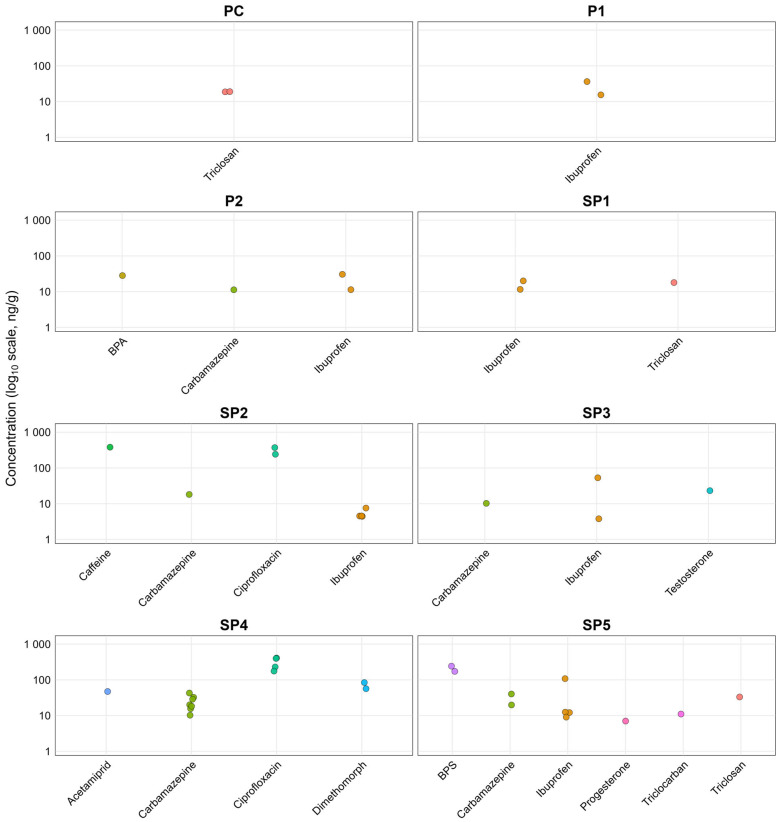
Jitter plots of CEC concentration (ng/g) in tomato samples across all treatments; PC: Peat substrate; P1: Peat substrate + CEC (0.3 mg/kg); P2: Peat substrate + CEC (3.5 mg/kg); SP1: Peat substrate + 45g of dried sludge; SP2: Peat substrate + 545 g of dried sludge; SP3: Peat substrate + 45 g of dried sludge + CEC (0.3 mg/kg); SP4: Peat substrate + 545 g of dried sludge + CEC (3.5 mg/kg); SP5: Peat substrate + Anaerobically digested sludge (1:1, *w*/*w*).

Tomato fruits accumulated a range of CECs, with distinct profiles depending on the treatment and whether CEC spiking was applied (Figure 2 and Table S15). The highest concentrations were found for triclosan in PC (19 ng/g), ibuprofen in SP1 (20 ng/g), ciprofloxacin in SP2 (372 ng/g) and BPS in SP5 (241 ng/g). For spiked treatments, the highest concentrations were found for ibuprofen in P1 (30.5 ng/g), P2 (30.5 ng/g), and SP3 (53.1 ng/g) and for ciprofloxacin in SP4 (408 ng/g). The BCFs ranged from 0.006 to 35, where values > 1 indicate accumulation of CEC in tomato fruits (Table S16). The highest BCF values were obtained for SP1 (ibuprofen: 6.1, triclosan: 1.3), SP2 (caffeine: 35, carbamazepine: 1.6, ibuprofen: 0.06) and SP5 (BPS: 21, carbamazepine: 12, ibuprofen: 0.6, progesterone: 0.1, triclocarban: 0.7 and triclosan: 0.2). The higher number of CECs in fruits grown in SP5 compared to SP1 and SP2 may also be due to the effect of drying, which has been shown to reduce levels of CECs [83].

Within spiked treatments (P1, P2, SP3 and SP4), where different translocation factors were calculated for carbamazepine, translocation to leaves was over 100 times higher than in fruits, indicating that even in high contamination conditions, bioaccumulation in fruits was reduced compared to other plant parts. Differences between the concentrations of carbamazepine in SP4 were significantly higher compared to P2 (*p* = 0.02; effect size d = 1.9). The difference in CEC bioaccumulation observed between P2 and SP4, despite similar final CEC concentrations, may be attributed to the addition of sludge, as previously reported in the literature [15,19].

Triclosan and triclocarban have been studied previously in various crops, since they are consistently detected in sewage sludge and biosolids [84,85]. Among those studies that investigated tomatoes (Table 1), only Prosser et al. [29] were able to detect triclocarban (up to 5.2 ng/g), whereas in the present study, triclosan was quantified in SP1 (≤17.9 ng/g), and triclocarban was determined in SP5 (≤33.1 ng/g). Wu et al. [22] also studied carbamazepine and triclocarban in tomato grown in spiked biosolid-amended soil and found a higher amount of triclocarban than carbamazepine, which is opposite to the present study (SP4, carbamazepine: 42.7 ng/g; triclocarban: <LOQ). Another field study by Sabourin et al. [28] with dewatered biosolids application, detected only six out of 141 Pharmaceutical and Personal Care Products (PPCPs) in tomatoes, including testosterone, which was only quantified in tomatoes grown in spiked sludge (SP3; 23.2 ng/g), while they did not detect caffeine, carbamazepine, ciprofloxacin, ibuprofen and triclosan, which were quantified in SP1 and SP2.

Ibuprofen and diclofenac were also previously detected in tomatoes grown in spiked soil [86], with concentrations ≤ 95.2 ng/g for ibuprofen and 84.2 ng/g for diclofenac, respectively. However, diclofenac was not applicable in our study since it was not detected, whereas ibuprofen was quantified across six different treatments. Ibuprofen, a weak acid with a pKa of 4.9, is predominantly in its anionic form at the pH values of the tested media. Anions are more soluble compared to the protonated form of the molecule, which reduces sorption affinity onto organic matter and may enhance pore-water mobility [87]. Although CECs in anionic form are less prone to plant uptake, due to repellence from negative electrical potential at the cell membrane [88], other parameters and processes may also influence the uptake and translocation, including membrane permeability, ion trap, phloem transport, and sorption to proteins, as well as molecular weight, the pH around and inside the cell membranes and plant physiological characters (root lipid content and carbohydrates) [89,90].

Overall, the results demonstrate that uptake is not only associated with the concentration introduced into the substrate, but is also affected by potential interactions between the growing matrix (peat substrate and its combinations with sludge) and the compounds evaluated. These can affect their sorption, degradation, and metabolism within the plant system. Differences between this study and the available literature may be attributed to the use of a commercial peat substrate, which has different characteristics from soil.

### 3.4. Tomato Yield

The yield average (Table S17) between the treatments showed no significant difference (*p* > 0.05) in total weight or fruit number per replicate, likely due to limited and unbalanced replication between treatments and fertilised groups. When comparison was applied between pooled fertilised (F) and non-fertilised (NF) treatments, both yield and number of fruits had a significant difference between F and NF (higher in F, *p* = 0.009 and 0.03), while the presence or absence of CECs within treatments did not affect the yield or number of fruits (*p* = 0.7 and 0.8). Spearman’s rank correlation analysis showed a statistically significant positive correlation between the number of fruits collected and the yield per replicate (ρ = 0.96, *p* < 0.001). This finding suggests that differences in yield weight were primarily driven by differences in fruit number rather than individual fruit size.

Plants with sludge amendment generally showed stress symptoms, including leaf chlorosis and necrosis (Figure S4, left), which may result from the potentially toxic element content in sludge and its salinity, both of which can reduce water uptake and inhibit growth [15,91]. Although the conditions for ammonium nitrification in pots with sludge were favourable (average daily air temperature during the growth period was 22.5 °C, with sufficient substrate aeration and water content), there was enough nitrate for the growth and development of tomato plants.

**Figure 3:**
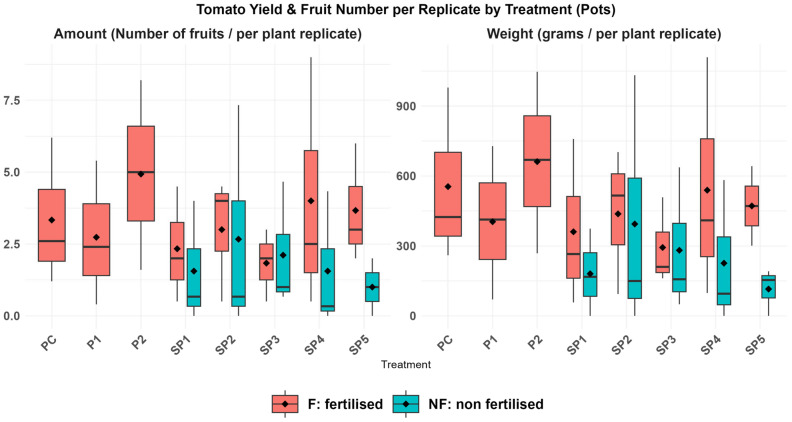
Boxplots of tomato yield and number of fruits per treatment, normalised per replicate.

Toxic symptoms in plants, which appeared in the leaves of those treated with sludge, were probably a consequence of the nitrification process in soil containing sludge, as nitrogen in sludge was mainly present as ammonium. In soil, the uptake of nitrate by plants is rapid, whereas the uptake of ammonium is slower. Ammonium binds to soil particles (clay and organic matter) and is slowly converted to nitrate over several days or weeks during nitrification by soil bacteria. During nitrification, N_2_O and NO are lost to the atmosphere, potentially causing toxic symptoms in plant leaves [92,93]. Notably, after adding fertiliser, plant growth visibly improved, and there was no significant difference in yield across all treatments (Figure 3).

### 3.5. Dietary Exposure to CECs and Health Risk Assessment for Tomato Fruits

The average chronic dietary exposure (mg kg^−1^ bw day^−1^) was estimated for adults, adolescents, and toddlers in Slovenia under both average and high tomato consumption, using fresh-weight concentrations from each treatment (Tables S18 and S19). Risk characterisation was performed by comparing each EDI with the corresponding health-based reference values.

The data obtained show that estimated daily intakes are below the risk threshold for most compounds quantified across different treatments (HQ < 1). Risk was identified only for two bisphenols. Specifically, BPA exceeded the TDI for all age groups and consumption margins in high-spiked treatments (P2) (HQ: 1.3–18). During the mixture of sludge with peat substrate (SP5), only BPS exceeded the tolerable daily intake (HQ: 11–153). Due to the lack of health-based reference values for BPS, the suggested TDI by EFSA for BPA (0.2 ng kg^−1^ bw day^−1^) was used as a conservative comparison and due to structural similarities. However, for both bisphenols, the respective LOQs exceed the established EFSA TDI. When a bisphenol result is reported as <LOQ, it does not mean the concentration is at or below the EFSA-TDI. Instead, it suggests that the actual value may lie anywhere from zero to a range that could still imply risk. Although this may be considered a limitation, the method was designed to quantify CECs from different groups rather than to achieve trace-level sensitivity for bisphenols; thus, the degree of potential risk for this group cannot be precisely specified.

For the detected pesticide residues, both chronic and acute dietary exposure were calculated, and the latter was compared with the appropriate toxicological reference values. The acute exposure assessment models a high-intake event, and all estimated intakes were below the risk thresholds. This outcome was expected, as acetamiprid is an authorised pesticide in the EU for use on tomatoes, with an MRL of 0.06 mg/kg [94], whereas dimethomorph is no longer authorised and its MRL has been set at 0.05 mg/kg (LOQ) [95].

In all other treatments, no dietary exposure risk was identified for the compounds quantified in edible parts, including pharmaceuticals (carbamazepine, ciprofloxacin, ibuprofen), personal care products (triclosan, triclocarban), hormones (testosterone, progesterone), and stimulants (caffeine). However, this assessment includes only a representative target list, while metabolites and transformation products of the selected CEC could pose risks through dietary intake; further studies are needed to address risks and synergistic effects deriving from mixtures.

### 3.6. Ecological Risk Assessment of CECs in Peat Substrate

The potential ecological risks posed by each CEC within the growth media of different treatments, as collected at the end of the experiment, were evaluated using RQ values, which are provided in Table S20. Although the described experiment was performed in pots, where substrate was handled and disposed of in accordance with Slovenian waste management legislation after the finalisation of a growing season [31], the ecological risk assessment is considered important since the results can be extrapolated for similar applications to field-grown tomatoes, especially for scenarios with consecutive sludge applications within the years. Apart from CECs’ persistence in soil following the application of sludge, another concern with the practice is the potential leaching of compounds into surface water sources and groundwater [96]. However, this aspect was not evaluated because the experimental design included pots in which leachate from individual pots was not collected. Although the method to calculate PNEC for soil may lead to overestimation, high ecological risk (RQ > 1) was found across different treatments. In control samples, two compounds exceeded the threshold: benzophenone and triclocarban.

Benzophenone had similar RQ values (1.5) when sludge was introduced at a lower dose, where triclocarban was below LOQ. In the higher sludge dose (SP2), risks were identified for triclosan, triclocarban, and tonalide, while the RQ value for benzophenone remained below 1 (0.62), indicating a medium risk. However, the evaluation of the peat substrate/dewatered sludge mix revealed the highest RQ values among the treatments where CEC was not deliberately applied. In this case, high risk was identified for BPA (18.9), benzophenone (2.5), estrone (3.7), ibuprofen (238), tonalide (296), triclocarban (770) and triclosan (131).

The limited identification of CEC and the reduced risk associated with using dried sludge amendment compared to anaerobically digested sludge are consistent with the literature. For example, torrefaction of sludge before amendment is a process that significantly reduces the CEC content in sludge and biosolids, thereby limiting their presence in the receiving substrate and their uptake by plants [97,98]. Triclosan and triclocarban are both persistent in sludge-amended soils and may pose a risk to soil health, as they can disrupt microbial communities and inhibit organic matter decomposition by suppressing microorganisms [99,100]. In spiked treatments, either spiked peat substrate or spiked sludge, RQs exceeded safety limits for most of the compounds quantified. This finding indicates that although the levels of CECs significantly decreased from the beginning to the end of the experiment, the risk remained high for most CECs (16 out of 22 CECs) in SP4 after 117 days. Although the initially applied concentrations of CECs were higher than would be expected from sludge amendment, the results highlight the persistence of several CECs, which can affect the soil ecosystem. Their presence can impact soil processes, and the build-up of antibiotic resistance is a major concern [101,102].

### 3.7. Limitations and Future Work

While findings provide valuable insights into the uptake of CECs in tomato plants following sludge application, several limitations should be acknowledged. The results are based on a controlled greenhouse pot experiment and may therefore not fully reflect field conditions or commercial production. Applying sludge repeatedly over several growing seasons could also lead to the build-up of persistent CECs in the soil, thereby increasing the likelihood of their uptake and translocation to the fruits, and raising the overall risk from sludge reuse; however, this was not assessed in the current single-season study. Moreover, the uptake of CECs in plants can be influenced by variations in sludge composition and soil characteristics, limiting broader generalisation. Analytical limitations should be considered, since compounds just below the LOQ may still be present and pose potential health risks. Additionally, metabolites and transformation products of the selected CECs were not included in the analysis, which may have potentially underestimated overall exposure.

For ecological risk assessment, PNEC values derived using the equilibrium partitioning method serve as a screening tool but may lead to over- or underestimations. In the dietary exposure assessment, a deterministic (point-estimate) approach was applied, which does not account for chemical mixtures and potential synergistic effects. Although a probabilistic exposure model would provide a more realistic assessment, it was not applied due to insufficient data to characterise the distribution of individual chemicals properly.

## 4. Conclusions

This study looked at the uptake of CECs by tomato plants grown in sludge-amended peat. Several CECs were determined in the amended substrate, with more compounds quantified at the higher amended rate. A higher number of compounds was also quantified when non-dried anaerobically digested sludge was added. In all treatments, RCF was <1, indicating that the concentration in the roots was lower than in the peat substrate. For carbamazepine, the only compound detected in each tissue, translocation from roots to leaves was highest, followed by stems and fruit. BCFs were >1 in dried sludge for caffeine, carbamazepine, ibuprofen, and triclosan, while for non-dried sludge, BCFs were >1 for BPS and carbamazepine. Although adding sludge affected plant health, neither sludge nor CECs had a measurable effect on tomato yield. According to dietary exposure estimates, a risk was only identified for BPS when non-dried anaerobically digested sludge was applied. In terms of ecological risk, the study showed risk quotients > 1 for 7 out of 27 CECs in treatments that included sludge, which could have implications if sludge is applied repeatedly in the field. Future work should extend to monitoring non-targeted CEC residues, including their stable transformation products and address them in human and ecological risk assessment.

## Figures and Tables

**Table 1:** Literature on uptake studies, including the growth of tomatoes in sludge/biosolids.

Compounds Studied	Plants Studied	Treatment	Compounds in Tomato Fruits	Refs
Carbamazepine, 10,11-Epoxide-carbamazepine, Dihydroxy-carbamazepine	Tomato, lettuce, wheat	Composted biosolids	Carbamazepine, Dihydroxy-carbamazepine	[17]
Synthetic musks and UV filters	Tomato	Composted biosolids (spiked up to 500 ng/g)	15 musks and UV filters, including tonalide & benzophenone	[21]
Carbamazepine, Diphenhydramine, Triclocarban	Pepper, tomato, collard, lettuce, radish	Spiked biosolids (100 ng/g)	Diphenhydramine, Triclocarban	[22]
30 PFASs	Tomato	Biosolids	PFBA; PFPeA; PFHxA, PFOA	[23]
PFASs, PBDEs and Dechloranes	Spinach, tomato	Anaerobically digested thermally dried sludge	PFASs, PBDEs and Dechloranes	[24]
12 PFASs	Lettuce, tomato, corn	Biosolids	PFBA, PFPeA, PFHxA, PFHpA, PFOA, PFBS, PFHxS	[27]
118 PPCPs or PPCP TPs, 6 parabens, and 17 hormones or hormone TPs	Barley, carrot, potato, sweet corn, tomato	Dewatered municipal biosolids	Atenolol, DEET, Minocycline, Sulfamerazine, Testosterone, Trimethoprim	[28]
Triclosan and Triclocarban	Carrot, cucumber, tomato, green bell pepper	Dewatered anaerobically digested biosolids	Triclocarban	[29]

**Table 2:** Treatments included in the uptake experiment.

Abbreviation	Treatment	Addition of CEC
PC	Peat substrate (control)	-
P1	Peat substrate + CEC	0.3 mg/kg
P2	Peat substrate + CEC	3.5 mg/kg
SP1	Peat substrate + 45 g of dried sludge	-
SP2	Peat substrate + 545 g of dried sludge	-
SP3	Peat substrate + 45 g of dried sludge + CEC	0.3 mg/kg
SP4	Peat substrate + 545 g of dried sludge + CEC	3.5 mg/kg
SP5	Peat substrate + Anaerobically digested sludge (1:1, *w*/*w*)	-

## Data Availability

The raw data supporting the conclusions of this article will be made available by the authors on request.

## Supplementary Materials

The following supporting information can be downloaded at https://www.mdpi.com/article/10.3390/toxics13121013/s1. Table S1: Names, CAS numbers, molecular formula, octanol–water partition coefficient (log Kow), pKa, organic carbon partition coefficient (Koc) and molecular weights (MW) of the investigated contaminants of emerging concern (CECs) derived from PubChem, DrugBank and Human Metabolome Database [1]; Table S2: Characteristics of peat substrate; Table S3: Analysis of nutrients in dried sludge; Figure S1 (a) Schematic showing the randomised planting scheme including different replicates (I-V), and (b) photo showing pots with 5-week-old tomato plants and the layout of the drip irrigation system; Figure S2 (a) Anaerobically digested sludge, (b) Dried sludge and (c) Spiked sludge before solvent evaporation; Figure S3: Temperature and solar radiation measurements across the experimental period (May–September 2022, 117 days); Figure S4: Visual difference between pot amended with a high amount of sludge (SP2, left) and control (P, right); Table S4: Validation data on recovery (REC), matrix effect (ME), repeatability (REP) for high and low point, LOD, LOQ and R^2^ for (a) peat substrate matrix and (b) sludge matrix. Table S5: Consumption rates for tomatoes in Slovenia, acquired from EFSA Food consumption statistics; Table S6: Health-based reference values, including Acceptable Daily Intake (ADI), Acute Reference Dose (ARfD), Tolerable Daily Intake (TDI) or No Observed Adverse Effect Level (NOAEL) for individual CEC; Table S7: Predicted environmental concentrations (PNEC) of each CEC in soil based on the PNEC of freshwater; Table S8: CEC concentration (ng/g) in dried sludge used in the experiments; Table S9: Concentration of CEC in peat substrate at the beginning of the experiment, expressed in ng/g d.w.; Table S10: Concentration of CEC in peat substrate at the end of the experiment, expressed in ng/g d.w.; Table S11: Analysis of substrate samples at the end of the experiment; Figure S5: Boxplots showing CEC concentrations at month 0 and month 3 for P1; Figure S6: Boxplots showing CEC concentrations at month 0 and month 3 for P2; Figure S7: Boxplots showing CEC concentrations at month 0 and month 3 for SP3; Figure S8: Boxplots showing CEC concentrations at month 0 and month 3 for SP4; Table S12: Month 0 vs. Month 3 comparisons per compound and treatment; Table S13: Average concentration of CEC in plant parts, expressed in ng/g d.w.; Table S14: Root Concentration Factor (RCF) calculated based on peat substrate measurement; Table S15: Concentration of CEC quantified in tomato samples, including composite samples per plant (1–5) and composite samples per harvest season (A–C); Table S16: Bioconcentration Factor (BCF) calculated based on peat substrate concentration; Table S17: Tomato yield production from different treatments and seasons; Table S18: Estimated Daily Intake (EDI) as mg/kg per body weight per day; Table S19: Hazard Quotients for CECs in tomato fruits; Table S20: Ecological risk assessment results based on Risk Quotient (RQ). [32,33,34,35,36,39,41,42,43,44,45,46,47,48,49,50,51,52,53,54,55,58].

## Abbreviations

The following abbreviations are used in this manuscript:

CECs	Contaminants of Emerging Concern
PPCPs	Pharmaceutical and Personal Care Products
PFASs	Per- and Polyfluoroalkyl Substances
PBDEs	Polybrominated Diphenyl Ethers
WWTP	Wastewater Treatment Plant
EDI	Estimated Daily Intake
ADI	Acceptable Daily Intake
TDI	Tolerable Daily Intake
NOAEL	No Observed Adverse Effect Level
MEC	Measured Environmental Concentration
PNEC	Predicted No-Effect Concentration
EPM	Equilibrium Partitioning Method
BCF	Bioconcentration Factor
RCF	Root Concentration Factor
TF	Translocation Factor

## References

[1] Liu H. (2016). Achilles Heel of Environmental Risk from Recycling of Sludge to Soil as Amendment: A Summary in Recent Ten Years (2007–2016). Waste Manag..

[2] Singh R.P., Agrawal M. (2008). Potential Benefits and Risks of Land Application of Sewage Sludge. Waste Manag..

[3] European Environment Agency, Long-Term Impacts of Sludge Spreading on Agricultural Land (Signal) | European Zero Pollution Dashboards. https://www.eea.europa.eu/en/european-zero-pollution-dashboards/indicators/long-term-impacts-of-sludge-spreading-on-agricultural-land.

[4] Huygens D., García-Gutierrez P., Orveillon G., Schillaci C., Delre A., Orgiazzi A., Wojda P., Tonini D., Jones A., Pistocchi A. (2022). Screening Risk Assessment of Organic Pollutants and Environmental Impacts from Sewage Sludge Management. Study to Support Policy Development on the Sewage Sludge Directive (86/278/EEC).

[5] European Commission (2022). Implementation Report of the Sewage Sludge Directive 86/278/EEC.

[6] European Commission (1986). Council Directive 86/278/EEC of 12 June 1986 on the Protection of the Environment, and in Particular of the Soil, When Sewage Sludge Is Used in Agriculture.

[7] Collivignarelli M.C., Abbà A., Frattarola A., Miino M.C., Padovani S., Katsoyiannis I., Torretta V. (2019). Legislation for the Reuse of Biosolids on Agricultural Land in Europe: Overview. Sustainability.

[8] Koumoulidis D., Varvaris I., Pittaki Z., Hadjimitsis D. (2024). Sewage Sludge in Agricultural Lands: The Legislative Framework in EU-28. Sustainability.

[9] Kelessidis A., Stasinakis A.S. (2012). Comparative Study of the Methods Used for Treatment and Final Disposal of Sewage Sludge in European Countries. Waste Manag..

[10] Basri S., Oruganti R.K., Panda T.K., Bhattacharyya D. (2025). Beyond Conventional Approaches: Sustainable Valorization of Sewage Sludge—Challenges and Opportunities. Sustain. Mater. Technol..

[11] Lucia C., Badalucco L., Corsino S.F., Galati A., Iovino M., Muscarella S.M., Paliaga S., Torregrossa M., Laudicina V.A. (2025). Management and Valorisation of Sewage Sludge to Foster the Circular Economy in the Agricultural Sector. Discov. Soil..

[12] Collivignarelli M.C., Canato M., Abbà A., Carnevale Miino M. (2019). Biosolids: What Are the Different Types of Reuse?. J. Clean. Prod..

[13] Bair D.A., Anderson C.G., Chung Y., Scow K.M., Franco R.B., Parikh S.J. (2020). Impact of Biochar on Plant Growth and Uptake of Ciprofloxacin, Triclocarban and Triclosan from Biosolids. J. Environ. Sci. Health B.

[14] Vehar A., Kovačič A., Hvala N., Škufca D., Levstek M., Stražar M., Žgajnar Gotvajn A., Heath E. (2022). An Assessment of Mass Flows, Removal and Environmental Emissions of Bisphenols in a Sequencing Batch Reactor Wastewater Treatment Plant. Molecules.

[15] Kodešová R., Klement A., Golovko O., Fér M., Kočárek M., Nikodem A., Grabic R. (2019). Soil Influences on Uptake and Transfer of Pharmaceuticals from Sewage Sludge Amended Soils to Spinach. J. Environ. Manag..

[16] Li M., Ding T., Wang H., Wang W., Ye Q., Li J. (2020). Biosolids Inhibit Uptake and Translocation of 14C-Carbamazepine by Edible Vegetables in Soil. Environ. Sci. Pollut. Res..

[17] Ben Mordechay E., Tarchitzky J., Chen Y., Shenker M., Chefetz B. (2018). Composted Biosolids and Treated Wastewater as Sources of Pharmaceuticals and Personal Care Products for Plant Uptake: A Case Study with Carbamazepine. Environ. Pollut..

[18] Xu J., Wang Y., Zhang Q., Sun H., Zhang W. (2023). Uptake and Enantiomeric Selectivity of β-Blockers in Lettuce (*Lactuca sativa* L.) and Tomato (*Lycopersicon esculentum* M.) in Soil–Pot Culture. J. Agric. Food Chem..

[19] Berthod L., Roberts G., Sharpe A., Whitley D.C., Greenwood R., Mills G.A. (2016). Effect of Sewage Sludge Type on the Partitioning Behaviour of Pharmaceuticals: A Meta-Analysis. Environ. Sci..

[20] Mejías C., Martín J., Santos J.L., Aparicio I., Alonso E. (2021). Occurrence of Pharmaceuticals and Their Metabolites in Sewage Sludge and Soil: A Review on Their Distribution and Environmental Risk Assessment. Trends Environ. Anal. Chem..

[21] Ramos S., Homem V., Santos L. (2021). Uptake and Translocation of UV-Filters and Synthetic Musk Compounds into Edible Parts of Tomato Grown in Amended Soils. Sci. Total Environ..

[22] Wu C., Spongberg A.L., Witter J.D., Sridhar B.B.M. (2012). Transfer of Wastewater Associated Pharmaceuticals and Personal Care Products to Crop Plants from Biosolids Treated Soil. Ecotoxicol. Environ. Saf..

[23] Broadbent E., Gravesen C., Choi Y.J., Lee L., Wilson P.C., Judy J.D. (2025). Effects of Drinking Water Treatment Residual Amendments to Biosolids on Plant Uptake of Per- and Polyfluoroalkyl Substances. J. Environ. Qual..

[24] Navarro I., de la Torre A., Sanz P., Porcel M.Á., Pro J., Carbonell G., de los Ángeles Martínez M. (2017). Uptake of Perfluoroalkyl Substances and Halogenated Flame Retardants by Crop Plants Grown in Biosolids-Amended Soils. Environ. Res..

[25] Kinney C.A., Vanden B. (2020). Heuvel Translocation of Pharmaceuticals and Personal Care Products after Land Application of Biosolids. Curr. Opin. Environ. Sci. Health.

[26] Bolesta W., Głodniok M., Styszko K. (2022). From Sewage Sludge to the Soil—Transfer of Pharmaceuticals: A Review. Int. J. Environ. Res. Public Health.

[27] Blaine A.C., Rich C.D., Hundal L.S., Lau C., Mills M.A., Harris K.M., Higgins C.P. (2013). Uptake of Perfluoroalkyl Acids into Edible Crops via Land Applied Biosolids: Field and Greenhouse Studies. Environ. Sci. Technol..

[28] Sabourin L., Duenk P., Bonte-Gelok S., Payne M., Lapen D.R., Topp E. (2012). Uptake of Pharmaceuticals, Hormones and Parabens into Vegetables Grown in Soil Fertilized with Municipal Biosolids. Sci. Total Environ..

[29] Prosser R.S., Lissemore L., Topp E., Sibley P.K. (2014). Bioaccumulation of Triclosan and Triclocarban in Plants Grown in Soils Amended with Municipal Dewatered Biosolids. Environ. Toxicol. Chem..

[30] Boguniewicz-Zablocka J., Klosok-Bazan I., Capodaglio A.G. (2021). Sustainable Management of Biological Solids in Small Treatment Plants: Overview of Strategies and Reuse Options for a Solar Drying Facility in Poland. Environ. Sci. Pollut. Res..

[31] Ministry of the Environment and Spatial Planning (2008). Decree on Waste Management, Official Gazette RS No. 34/2008: Regulation on the Loading of Soil Through the Introduction of Waste.

[32] (2006). Soil Quality—Pretreatment of Samples for Physico-Chemical Analysis.

[33] (1995). Soil Quality—Determination of Organic and Total Carbon after Dry Combustion (Elementary Analysis).

[34] (1998). Soil Quality—Determination of Total Nitrogen Content by Dry Combustion (“Elemental Analysis”).

[35] Egnér H., Riehm H., Domingo W.R. (1960). Untersuchungen Uber Die Chemishe Boden-Analyse Als Grundlage Fur Die Beurteilung Des Nahrsoffzustandes Der Boden. II. Chemiche Extraktionsmethoden Zur Phosphor Und Kalimbestimmung. K. Lantbrukshögskolans Ann..

[36] (2021). Soil, Treated Biowaste and Sludge—Determination of PH.

[37] Baumgarten A., Almesberger M., Eschlböck K., Greimel J., Hamedinger S., Hoffmann G., Hofmair W., Keferböck J., Kovats H.G., Mayer J. (2008). Richtlinien Für Die Sachgerechte Düngung Im Garten-Und Feldgemüsebau.

[38] Meteo.Si—Uradna Vremenska Napoved Za Slovenijo—Državna Meteorološka Služba RS—Povprečne Mesečne Vrednosti Evapotranspiracije v Obdobju 1971–2000. https://meteo.arso.gov.si/met/sl/agromet/period/etp/.

[39] Andreasidou E., Kovačič A., Manzano-Sánchez L., Heath D., Kosjek T., Pintar M., Maršič N.K., Blaznik U., Fernández-Alba A.R., Hernando M.D. (2025). Uptake of Emerging Contaminants in Tomato Plants: A Field Study on Treated Wastewater Reuse. Environ. Int..

[40] Andreasidou E., Martello L., Heath D., Bikiaris D.N., Lambropoulou D.A., Heath E. (2025). Synthesis and Evaluation of a New Acrylic Copolymer for Dispersive Solid-Phase Microextraction of Organic Contaminants from Urban Wastewater. Microchem. J..

[41] European Commission (2021). DG-SANTE Guidance Document on the Analytical Quality Control and Method Validation Procedures for Pesticide Residues in Food and Feed. No SANTE 11312/2021 V2.

[42] Wenzl T., Haedrich J., Schaechtele A., Robouch P., Stroka J., Burdaspal P., Kapp T., Amlund H., Jensen U., Patriarca M. (2016). Guidance Document on the Estimation of LOD and LOQ for Measurements in the Field of Contaminants in Feed and Food.

[43] Fučík J., Jašek V., Hamplová M., Navrkalová J., Zlámalová Gargošová H., Mravcová L. (2024). Assessing Lettuce Exposure to a Multi-Pharmaceutical Mixture in Soil: Insights from LC-ESI-TQ Analysis and the Impact of Biochar on Pharmaceutical Bioavailability. ACS Omega.

[44] EFSA Food Consumption Statistics for FoodEx2: Level 7 | EFSA. https://www.efsa.europa.eu/en/microstrategy/foodex2-level-7.

[45] Food and Agriculture Organization of the United Nations (2009). World Health Organization Principles and Methods for the Risk Assessment of Chemicals in Food.

[46] European Food Safety Authority (2025). Review of the Methodology Used for the Assessment of the Short-Term (Acute) Dietary Exposure to Pesticide Residues in Food (IESTI Methodology). EFSA J..

[47] Lambré C., Barat Baviera J.M., Bolognesi C., Chesson A., Cocconcelli P.S., Crebelli R., Gott D.M., Grob K., Lampi E., Mengelers M. (2023). Re-Evaluation of the Risks to Public Health Related to the Presence of Bisphenol A (BPA) in Foodstuffs. EFSA J..

[48] FitzGerald R., Van Loveren H., Civitella C., Castoldi A.F., Bernasconi G. (2020). Assessment of New Information on Bisphenol S (BPS) Submitted in Response to the Decision 1 under REACH Regulation (EC) No 1907/2006. EFSA Support. Publ..

[49] Hernandez-Jerez A., Coja T., Paparella M., Price A., Henri J., Focks A., Louisse J., Terron A., Binaglia M., Guajardo I.M. (2024). Statement on the Toxicological Properties and Maximum Residue Levels of Acetamiprid and Its Metabolites. EFSA J..

[50] EFSA Panel on Dietetic Products, Nutrition and Allergies (NDA) (2015). Scientific Opinion on the Safety of Caffeine. EFSA J..

[51] Scientific Opinion on “Draft Environmental Quality Standards for Priority Substances under the Water Framework Directive”—Carbamazepine—European Commission. https://health.ec.europa.eu/publications/scientific-opinion-draft-environmental-quality-standards-priority-substances-under-water-framework-6_en.

[52] Khan U., Nicell J. (2015). Human Health Relevance of Pharmaceutically Active Compounds in Drinking Water. AAPS J..

[53] Alvarez F., Arena M., Auteri D., Binaglia M., Castoldi A.F., Chiusolo A., Colagiorgi A., Colas M., Crivellente F., De Lentdecker C. (2023). Peer Review of the Pesticide Risk Assessment of the Active Substance Dimethomorph. EFSA J..

[54] World Health Organization Evaluations of the Joint FAO/WHO Expert Committee on Food Additives (JECFA)—Progesterone. https://apps.who.int/food-additives-contaminants-jecfa-database/Home/Chemical/3580.

[55] Scientific Committee on Consumer Safety Scientific (2023). Advice on the Safety of Triclocarban and Triclosan as Substances with Potential Endocrine Disrupting Properties in Cosmetic Products.

[56] Kumar A., Xagoraraki I. (2010). Human Health Risk Assessment of Pharmaceuticals in Water: An Uncertainty Analysis for Meprobamate, Carbamazepine, and Phenytoin. Regul. Toxicol. Pharmacol..

[57] European Chemicals Agency (2008). Guidance on Information Requirements and Chemical Safety Assessment Chapter R.10: Characterisation of Dose [Concentration]-Response for Environment Guidance for the Implementation of REACH.

[58] NORMAN Ecotoxicology Database. https://www.norman-network.com/nds/ecotox/lowestPnecsIndex.php?checkSelect=1.

[59] European Chemicals Agency (2012). Guidance on Information Requirements and Chemical Safety Assessment Chapter R.16: Environmental Exposure Estimation.

[60] Fernandes R., Necci A., Krausmann E. (2022). Model(s) for the Dispersion of Hazardous Substances in Floodwaters for RAPID-N.

[61] Van Beelen P., Verbruggen E.M.J., Peijnenburg W.J.G.M. (2003). The Evaluation of the Equilibrium Partitioning Method Using Sensitivity Distributions of Species in Water and Soil. Chemosphere.

[62] Hernando M.D., Mezcua M., Fernández-Alba A.R., Barceló D. (2006). Environmental Risk Assessment of Pharmaceutical Residues in Wastewater Effluents, Surface Waters and Sediments. Talanta.

[63] The R Core Team (2023). R: A Language and Environment for Statistical Computing.

[64] Arachchige Chamila Samarasinghe S.V., Krishnan K., Aitken R.J., Naidu R., Megharaj M. (2021). Persistence of the Parabens in Soil and Their Potential Toxicity to Earthworms. Environ. Toxicol. Pharmacol..

[65] Gonzales I., Sengupta R., Mani S., Correa-Soto C.E., Bejgum B., Alvarez-Nunez F., Kiang Y.H. (2025). Understanding the Sorption of Paraben on Plastics Using Molecular Dynamics Simulations. Int. J. Pharm..

[66] Litz N.T., Müller J., Böhmer W. (2007). Occurrence of Polycyclic Musks in Sewage Sludge and Their Behaviour in Soils and Plants. Part 2: Investigation of Polycyclic Musks in Soils and Plants. J. Soils Sediments.

[67] Yun H., Liang B., Kong D., Li X., Wang A. (2020). Fate, Risk and Removal of Triclocarban: A Critical Review. J. Hazard. Mater..

[68] Miao L., Chen S., Yang H., Hong Y., Sun L., Yang J., Sun G., Liu Y., Li C., Zang H. (2023). Enhanced Bioremediation of Triclocarban-Contaminated Soil by Rhodococcus Rhodochrous BX2 and Pseudomonas Sp. LY-1 Immobilized on Biochar and Microbial Community Response. Front. Microbiol..

[69] Verlicchi P., Zambello E. (2015). Pharmaceuticals and Personal Care Products in Untreated and Treated Sewage Sludge: Occurrence and Environmental Risk in the Case of Application on Soil—A Critical Review. Sci. Total Environ..

[70] Caliman F.A., Gavrilescu M. (2009). Pharmaceuticals, Personal Care Products and Endocrine Disrupting Agents in the Environment—A Review. Clean.

[71] Gottschall N., Topp E., Metcalfe C., Edwards M., Payne M., Kleywegt S., Russell P., Lapen D.R. (2012). Pharmaceutical and Personal Care Products in Groundwater, Subsurface Drainage, Soil, and Wheat Grain, Following a High Single Application of Municipal Biosolids to a Field. Chemosphere.

[72] Yang Y.Y., Gray J.L., Furlong E.T., Davis J.G., Revello R.C., Borch T. (2012). Steroid Hormone Runoff from Agricultural Test Plots Applied with Municipal Biosolids. Environ. Sci. Technol..

[73] Cheng Z., Zhang C., Jiang W., Zhai W., Gao J., Wang P. (2022). Effects of the Presence of Triclocarban on the Degradation and Migration of Co-Occurring Pesticides in Soil. Environ. Pollut..

[74] Popoola L.T., Olawale T.O., Salami L. (2023). A Review on the Fate and Effects of Contaminants in Biosolids Applied on Land: Hazards and Government Regulatory Policies. Heliyon.

[75] Shao Y., Yang K., Jia R., Tian C., Zhu Y. (2018). Degradation of Triclosan and Carbamazepine in Two Agricultural and Garden Soils with Different Textures Amended with Composted Sewage Sludge. Int. J. Environ. Res. Public Health.

[76] Pérez D.J., Doucette W.J., Moore M.T. (2022). Contaminants of Emerging Concern (CECs) in Zea Mays: Uptake, Translocation and Distribution Tissue Patterns over the Time and Its Relation with Physicochemical Properties and Plant Transpiration Rate. Chemosphere.

[77] Madikizela L.M., Botha T.L., Kamika I., Msagati T.A.M. (2022). Uptake, Occurrence, and Effects of Nonsteroidal Anti-Inflammatory Drugs and Analgesics in Plants and Edible Crops. J. Agric. Food Chem..

[78] Christou A., Papadavid G., Dalias P., Fotopoulos V., Michael C., Bayona J.M., Piña B., Fatta-Kassinos D. (2019). Ranking of Crop Plants According to Their Potential to Uptake and Accumulate Contaminants of Emerging Concern. Environ. Res..

[79] Goldstein M., Shenker M., Chefetz B. (2014). Insights into the Uptake Processes of Wastewater-Borne Pharmaceuticals by Vegetables. Environ. Sci. Technol..

[80] Fu J., Li S., Yin S., Zhao X., Zhao E., Li L. (2024). Comprehensive Effects of Acetamiprid Uptake and Translocation from Soil on Pak Choi and Lettuce at the Environmental Level. Pestic. Biochem. Physiol..

[81] Miller E.L., Nason S.L., Karthikeyan K.G., Pedersen J.A. (2016). Root Uptake of Pharmaceuticals and Personal Care Product Ingredients. Environ. Sci. Technol..

[82] Chen X., Li Y., Jiang L., Jiang X., Hu B., Wang L., Zhang S., Zhang X. (2022). Uptake and Transport of Steroid Estrogens in Soil-Plant Systems and Their Dissipation in Rhizosphere: Influence Factors and Mechanisms. J. Hazard. Mater..

[83] Varjúová D., Staňová A.V., Grabicová K., Zakhar R., Bodík I. (2025). Thermal Methods of Sludge Processing—Are They Suitable for Pharmaceuticals and Illicit Drugs Removal from Sewage Sludge?. Biomass Convers. Biorefinery.

[84] Fu Q., Wu X., Ye Q., Ernst F., Gan J. (2016). Biosolids Inhibit Bioavailability and Plant Uptake of Triclosan and Triclocarban. Water Res..

[85] Shahmohamadloo R.S., Lissemore L., Prosser R.S., Sibley P.K. (2017). Evaluating the Effects of Triclosan on 3 Field Crops Grown in 4 Formulations of Biosolids. Environ. Toxicol. Chem..

[86] Kovacs E.D., Silaghi-Dumitrescu L., Kovacs M.H., Roman C. (2021). Determination of the Uptake of Ibuprofen, Ketoprofen, and Diclofenac by Tomatoes, Radishes, and Lettuce by Gas Chromatography–Mass Spectrometry (GC–MS). Anal. Lett..

[87] Durán-Álvarez J.C., Prado B., Ferroud A., Juayerk N., Jiménez-Cisneros B. (2014). Sorption, Desorption and Displacement of Ibuprofen, Estrone, and 17β Estradiol in Wastewater Irrigated and Rainfed Agricultural Soils. Sci. Total Environ..

[88] Wu X., Ernst F., Conkle J.L., Gan J. (2013). Comparative Uptake and Translocation of Pharmaceutical and Personal Care Products (PPCPs) by Common Vegetables. Environ. Int..

[89] Wei H., Tang M., Xu X. (2023). Mechanism of Uptake, Accumulation, Transport, Metabolism and Phytotoxic Effects of Pharmaceuticals and Personal Care Products within Plants: A Review. Sci. Total Environ..

[90] Trapp S., Shi J., Zeng L. (2023). Generic Model for Plant Uptake of Ionizable Pharmaceuticals and Personal Care Products. Environ. Toxicol. Chem..

[91] Khalid S., Shahid M., Natasha, Bibi I., Sarwar T., Shah A.H., Niazi N.K. (2018). A Review of Environmental Contamination and Health Risk Assessment of Wastewater Use for Crop Irrigation with a Focus on Low and High-Income Countries. Int. J. Environ. Res. Public Health.

[92] Sahrawat K.L. (2008). Factors Affecting Nitrification in Soils. Commun. Soil. Sci. Plant Anal..

[93] Wendeborn S. (2020). The Chemistry, Biology, and Modulation of Ammonium Nitrification in Soil. Angew. Chem. Int. Ed..

[94] European Commission (2025). Commission Regulation (EU) 2025/158 of 29 January 2025 Amending Annex II to Regulation (EC) No 396/2005 of the European Parliament and of the Council as Regards Maximum Residue Levels for Acetamiprid in or on Certain Products.

[95] European Commission (2024). Commission Implementing Regulation (EU) 2024/1207 of 29 April 2024 Concerning the Non-Renewal of the Approval of the Active Substance Dimethomorph, in Accordance with Regulation (EC) No 1107/2009 of the European Parliament and of the Council, and Amending Commission Implementing Regulation (EU) No 540/2011.

[96] Ghirardini A., Verlicchi P. (2019). A Review of Selected Microcontaminants and Microorganisms in Land Runoff and Tile Drainage in Treated Sludge-Amended Soils. Sci. Total Environ..

[97] Nang S., Mercl F., Košnář Z., Pierdonà L., Doležal P., Paul C.S., Tlustoš P. (2025). Torrefaction of Sewage Sludge: An Approach to Nutrient Recycling and Contaminant Reduction in Agriculture. Environ. Res..

[98] Mercl F., Košnář Z., Maršík P., Vojtíšek M., Dušek J., Száková J., Tlustoš P. (2021). Pyrolysis of Biosolids as an Effective Tool to Reduce the Uptake of Pharmaceuticals by Plants. J. Hazard. Mater..

[99] Agarwal D., Sharma K., Chaudhary H.D., Bhatt U., Soni V. (2025). Comprehensive Insights into Triclosan: Environmental Sources, Plant Uptake, Metabolism, Phytotoxicity, and Food Safety Risks. Next Sustain..

[100] Fu Q., Sanganyado E., Ye Q., Gan J. (2016). Meta-Analysis of Biosolid Effects on Persistence of Triclosan and Triclocarban in Soil. Environ. Pollut..

[101] Nguyen M.K., Lin C., Nguyen H.L., Hung N.T.Q., La D.D., Nguyen X.H., Chang S.W., Chung W.J., Nguyen D.D. (2023). Occurrence, Fate, and Potential Risk of Pharmaceutical Pollutants in Agriculture: Challenges and Environmentally Friendly Solutions. Sci. Total Environ..

[102] Petruzzelli G., Pezzarossa B., Pedron F. (2025). The Fate of Chemical Contaminants in Soil with a View to Potential Risk to Human Health: A Review. Environments.

