# Supplementary material for: Contaminants of Emerging Concern in Tomatoes Grown in Sludge-Amended Peat: Uptake, Translocation and Risk Assessment
Source: Toxics. 2025 Nov 22;13(12):1013. doi: 10.3390/toxics13121013 (PMC12737074; doi:10.3390/toxics13121013)
Supplement: Supplementary file 1 [file toxics-13-01013-s001.zip › toxics-3956916-supplementary.pdf]

SI-1 General information on investigated compounds

Table S1: Names, CAS numbers, molecular formula, octanol-water partition coefficient (log Kow), pKa, organic carbon partition coefficient (Koc) and molecular weights (MW) of the investigated contaminants of emerging concern (CECs) derived from PubChem, DrugBank and Human Metabolome Database [1].

Name	CAS number	Molecular formula	Log Kow	pKa	Koc (L/kg)	MW (g/mol)
17α-ethynylestradiol (EE2)	57-63-6	$C_{20}H_{24}O_2$	3.67	10.3	510	296.4
Acetamiprid	160430-64-8	$C_{10}H_{11}ClN_4$	0.80	0.7	132-267	222.6
Amoxicillin	26787-78-0	$C_{16}H_{19}N_3O_5S$	0.87	2.6	100	365.4
Azithromycin	83905-01-5	$C_{38}H_{72}N_2O_{12}$	4.02	8.5	3,100	749.0
Benzophenone	119-61-9	$C_{13}H_{10}O$	3.18	-7.5*	430, 517	182.2
beta-estradiol (E2)	50-28-2	$C_{18}H_{24}O_2$	4.01	10.4	30,000	272.4
Bisphenol F (4,4-BPF)	620-92-8	$C_{13}H_{12}O_2$	2.91	10.8	15,000	200.2
Bisphenol A (BPA)	80-05-7	$C_{15}H_{16}O_2$	3.43 - 3.32	9.6	636, 931	228.3
Bisphenol AF (BPAF)	2467-02-9	$C_{13}H_{10}Cl_2O_2$	4.26	9.2	760,000	336.2
Bisphenol S (BPS)	80-09-1	$C_{12}H_{10}O_4S$	1.65	8.2	160	250.3
Caffeine	58-08-2	$C_8H_{10}N_4O_2$	-0.07	14	71	194.2
Carbamazepine	298-46-4	$C_{15}H_{12}N_2O$	2.45	13.9	510	236.3
Ciprofloxacin	85721-33-1	$C_{17}H_{18}FN_3O_3$	0.28	6.09	61,000	331.3
Clarithromycin	81103-11-9	$C_{38}H_{69}NO_{13}$	3.16	8.99	150	748.0
Diclofenac as sodium salt	15307-79-6	$C_{14}H_{11}Cl_2NO_2$	4.51	4.15	245	296.1
Dimethomorph	110488-70-5	$C_{21}H_{22}ClNO_4$	2.63 (E) 2.73 (Z)	-1.3	5,690	387.9
Erythromycin	114-07-8	$C_{37}H_{67}NO_{13}$	3.06	12.4	570	733.9
Estrone (E1)	53-16-7	$C_{18}H_{22}O_2$	3.13	10.8	457-18,000	270.4
Ibuprofen	15687-27-1	$C_{13}H_{18}O_2$	3.97	4.91	3,400	206.8
Methylparaben	99-76-3	$C_8H_8O_3$	1.96	8.5	87	152.1
Naproxen	22204-53-1	$C_{14}H_{14}O_3$	3.18	4.15	330	230.3
Progesterone	57-83-0	$C_{21}H_{30}O_2$	3.87	18.9	2,800	314.5
Propylparaben	94-13-3	$C_{10}H_{12}O_3$	3.04	8.4	290	180.2
Testosterone	58-22-0	$C_{19}H_{28}O_2$	3.32	18.5	2,200	288.4
Tonalide	21145-77-7	$C_{18}H_{26}O$	5.70	16.3	19,000	258.4
Triclocarban	101-20-2	$C_{13}H_9Cl_3N_2O$	4.34	12.7	12,000	315.6
Triclosan	3380-34-5	$C_{12}H_7Cl_3O_2$	4.76	7.9	3,500	289.5

*refers to pKb for weak bases.

SI-2.1 Experimental Design

Analysis of total carbon and nitrogen and plant-available P and K

For dried sludge chemical analyses, samples were sieved to 2 mm [2]. Organic C and total N were determined by dry combustion [3,4] using an elemental analyser (Elementar vario MAX instrument, Germany). Plant-available phosphorus (P) and potassium (K) were determined after ammonium lactate extraction according to Egner-Riehm-Domingo [5]. Dried sludge pH was measured in a 1:2.5 (w/v) ratio of soil to 0.01 M CaCl2 suspension [6].

Table S2: Characteristics of peat substrate.

Characteristics	Result
Electrical conductivity (mS/m)	45
pH	5.5-6.5
Soil bulk density (g/L)	140 g/L
Organic matter content [%]	65%
Nutrients	
N (mg/L)	210
P_2O_5 (mg/L)	150
K_2O (mg/L)	270
Mg (mg/L)	100
S (total) (mg/L)	150

Table S3: Analysis of nutrients in dried sludge.

Characteristics	Result
pH in $CaCl_3$	6.4
P_2O_5 [mg/100g sample]	223.8
K_2O [mg/100g sample]	100
Total C [%]	28.17
Total N [%]	3.53

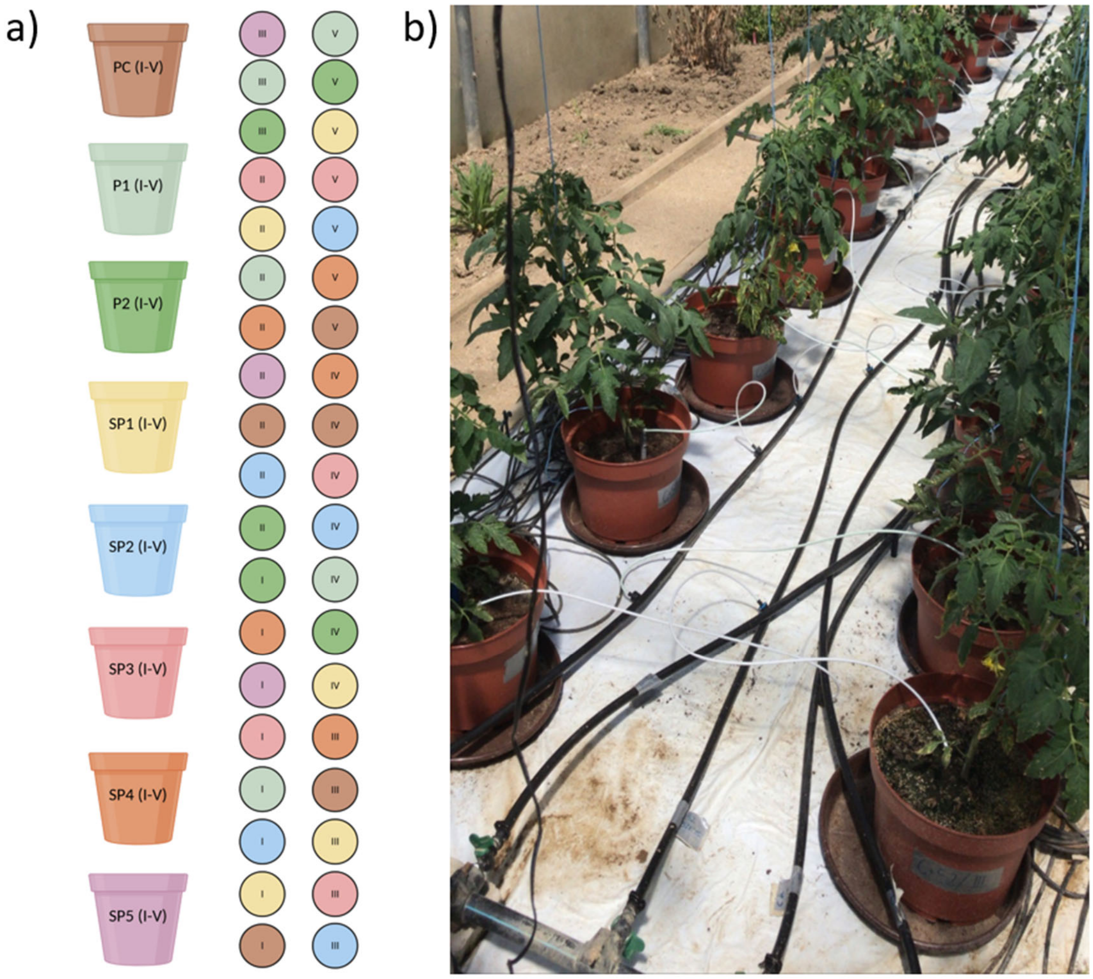

Figure S1. a) Schematic showing the randomised planting scheme including different replicates (I-V), and b) photo showing pots with 5-week-old tomato plants and the layout of the drip irrigation system.

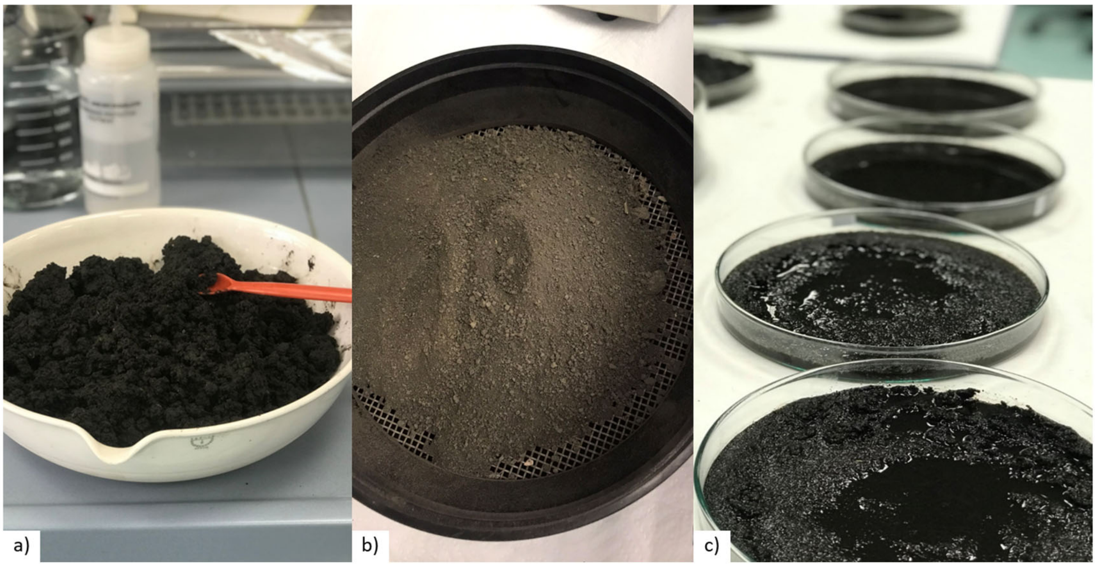

Fig. S2 a) Anaerobically digested sludge, b) Dried sludge and c) Spiked sludge before solvent evaporation.

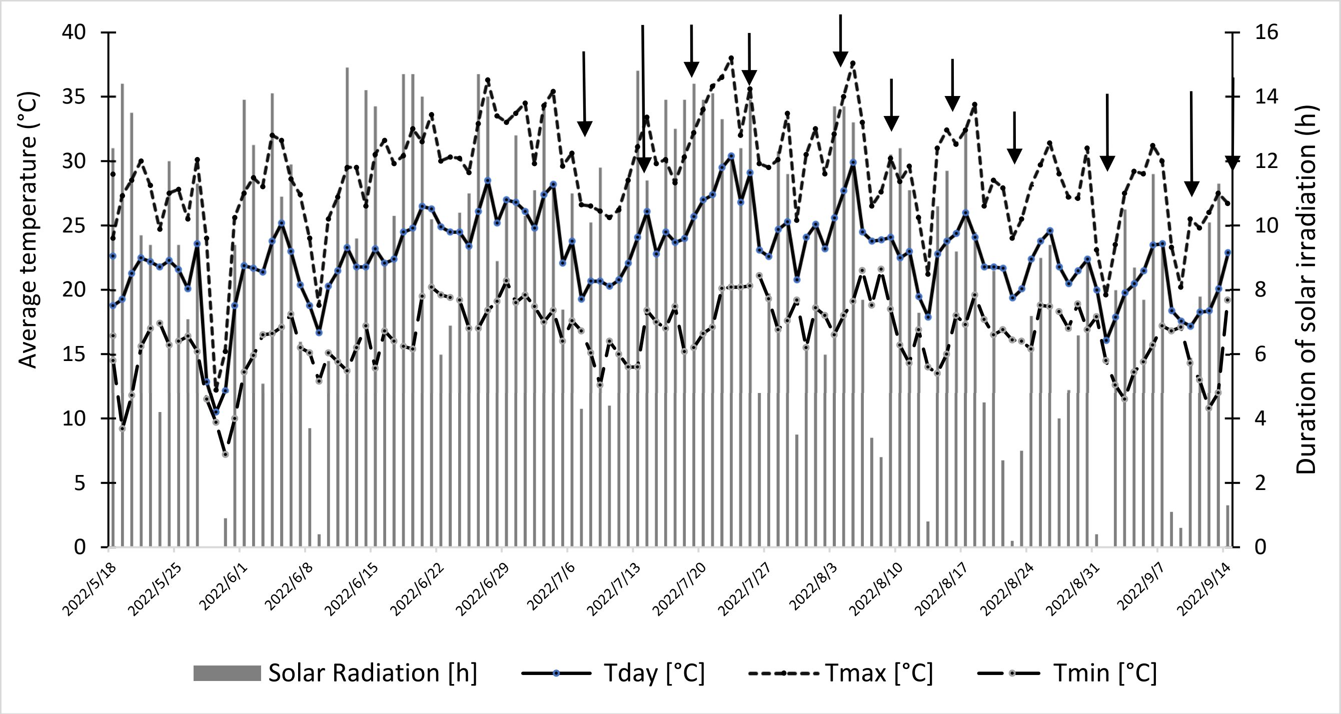

Fig. S3: Temperature and solar radiation measurements across the experimental period (May 2022-September 2022, 117 days).

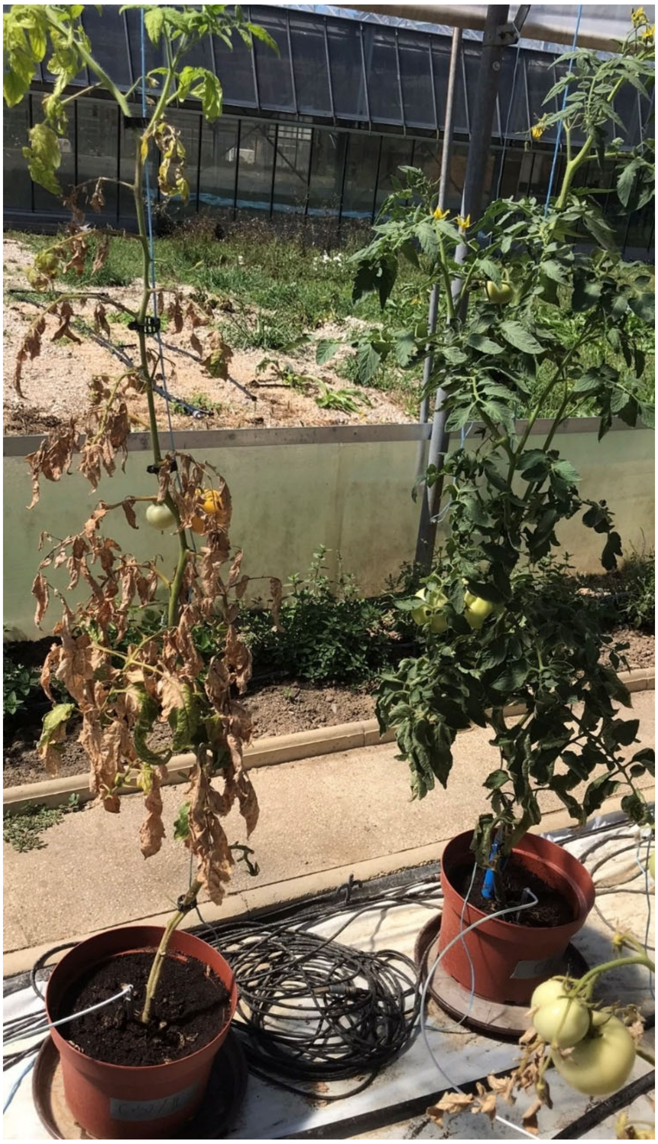

Fig. S4: Visual difference between pot amended with a high amount of sludge (SP2, left) and control (P, right).

SI-2.2 LC-MS/MS Analysis and Validation Results

LC-MS/MS data were processed using Analyst v1.6.3. For plant tissues (roots, stems, leaves), the method was transferred to a UHPLC system coupled to a 6500 QTRAP QqLIT-MS/MS (Sciex, Framingham, MA, USA), and data analysis was performed with SCIEX OS Software.

For quantification, 13 different isotopically labelled internal standards were used, considering factors such as ionization behaviour, structural similarities and retention time proximity. Internal standards and the corresponding target compounds (CECs) are listed below:

- BPA-d_{16}: BPA;
- $^{13}C_3$-caffeine: amoxicillin, ciprofloxacin, caffeine;
- Carbamazepine-d_{10}: carbamazepine, azithromycin, clarithromycin, erythromycin and testosterone;
- Clothianidin-d_3: acetamiprid;
- $^{13}C_6$-diclofenac: diclofenac, naproxen;
- ibuprofen-d_3: ibuprofen;
- $^{13}C_6$-methylparaben: methylparaben;
- $^{13}C_6$-propylparaben: propylparaben;
- Oxybenzone-d_5: benzophenone, dimethomorph, progesterone, tonalide;
- $^{13}C_{12}$-triclosan: triclosan, triclocarban;
- $^{13}C_2$-beta-estradiol: EE2, E2, E1;
- $^{13}C_{12}$-BPS: BPS;
- $^{13}C_{12}$-BPF: BPF, BPAF.

The linearity of methods was obtained by using procedural calibration curves, which can compensate for matrix effects and low extraction recoveries, especially where isotopically labelled standards are not available, prepared by spiking a series of blank test portions with selected amounts of analytes prior to extraction.

For peat substrate and sludge calibration curves, eight calibration points were included, with linear ranges of 2-1,000 ng/g. LOD was calculated by analysing blanks or pseudo-blanks by dividing the standard deviation of ten independent blank samples by the slope of the procedural calibration curve, multiplied by 3.9. LOQ was calculated as the LOD multiplied by 3.3, according to EURL Guidance Document [7].

All other validation parameters were measured at both low and high concentrations, within a selected range for each matrix: 10 and 250 ng/g for peat substrate samples, and 31.25 and 312.5 ng/g for sludge samples. Precision was expressed in method repeatability and calculated as the relative standard deviation (RSD) of three parallel samples (n = 3). Instrumental repeatability was determined as the RSD of three consecutive injections of the same solvent sample (n = 3) at a low and high level. Trueness was estimated as the method recovery. The acceptance criteria for validation required recoveries in the range of 30-140% when associated with RSDs ≤ 20%. Two compounds showed recoveries outside this range at one concentration level only: naproxen in the sludge matrix (157% at the low level) and triclocarban in the peat substrate (160% at the high level). However, their validation was accepted based on SANTE criteria: *"allows recoveries within the 60–140% range and recoveries to be within the range of the mean recovery +/- 2x RSD"* [8].

Table S4: Validation data on recovery (REC), matrix effect (ME), repeatability (REP) for high and low point, LOD, LOQ and R^2 for a) peat substrate matrix and b) sludge matrix.

a. Peat Substrate

Compound	REC H [%]	REC L [%]	ME H [%]	ME L [%]	REP H [%]	REP L [%]	LOD [ng/g]	LOQ [ng/g]	R^2
17α-ethynylestradiol	121	93.8	-17.7	33.6	19	4.5	1.5	4.9	0.998
Acetamiprid	107	89.1	-14.8	-3.85	18	1.8	7.6	25.1	0.999
Amoxicillin	NV	NV	NV	NV	NV	NV	NV	NV	NV
Azithromycin	104	73.7	-28.5	6.67	6.9	4.9	8.3	27.3	0.999
Benzophenone	96.6	89.1	135	473	22	6.2	7.8	25.7	0.996
BPA	118	98.3	-13.5	16.3	19	7.5	11.1	36.4	0.996
BPAF	113	90.3	-16.1	-37.1	15	18	1.0	3.3	0.999
BPF	112	96.3	-8.74	9.94	16	8.9	3.1	10.1	0.999
BPS	116	92.2	26.1	12.2	16	9.3	0.9	3.1	0.999
Caffeine	92.7	76.7	-25.4	7.95	20	9.5	7.8	25.7	0.999
Carbamazepine	101	87.9	2.38	6.44	18	6.5	7.8	25.8	0.978
Ciprofloxacin	NV	NV	NV	NV	NV	NV	NV	NV	NV
Clarithromycin	104	77.9	-27.8	0.32	6.4	8.8	9.1	26.9	0.998
Diclofenac	111	88.7	14.9	34.7	17	7.5	0.66	2.2	0.999
Dimethomorph	109	87.4	39.9	61.5	15	8.4	7.8	25.7	0.999
Erythromycin	80.3	62.3	-80.5	-73.8	18	10	9.7	32.1	0.998
Estradiol	120	101	-16.9	26.2	17	4.2	3.0	9.9	0.999
Estrone	121	88.5	-8.13	65.2	16	2.8	0.73	2.4	0.999
Ibuprofen	116	95.1	-11.6	-4.37	22	8.7	2.3	7.5	0.996
Methylparaben	109	107	-13.5	35.1	11	11	0.44	1.5	0.991
Naproxen	106	94.7	-33.6	-38.9	13	6.8	0.04	0.13	0.999
Progesterone	98.7	82.1	-38.6	-41.8	20	4.7	7.9	25.9	0.998
Propylparaben	110	91.6	-26.1	-38.1	11	5.3	0.09	0.29	0.991
Testosterone	105	88.6	-43.3	-36.7	15	3.8	7.7	25.5	0.999
Tonalide	109	89.4	-83.6	-19.5	3.2	17	7.8	25.7	0.986
Triclocarban	160	55.3	-87.3	-77.5	16	31	2.4	8.1	0.996
Triclosan	114	85.9	-10.5	23.1	15	10	5.2	17.2	0.995

[1]NV: not validated is used for values that did not meet the validation criteria.

b. Sludge

Compound	REC H [%]	REC L [%]	ME H [%]	ME L [%]	REP H [%]	REP L [%]	LOD [ng/g]	LOQ [ng/g]	R^2
17α-ethynylestradiol	100	81.5	-72.9	-57.9	2.7	7.3	7.5	24.6	0.994
Acetamiprid	92.7	102	-66.2	-66.6	6.5	7.3	15.6	51.5	0.998
Amoxicillin	NV	NV	NV	NV	NV	NV	NV	NV	NV
Azithromycin	81.9	106	-89.7	-93.0	14	7.4	39.0	128.7	0.931
Benzophenone	94.6	98.4	213	2,489	19	3.2	6.1	20.1	0.915
BPA	92.4	102	2,595	2,761	6.0	19	1,940	6,402	0.937
BPAF	96.2	97.8	-17.9	1.06	5.7	19	3.8	12.6	0.981
BPF	84.6	104	50.7	199	5.8	5.2	15.2	50.1	0.993
BPS	85.9	89.6	-44.5	-2.56	4.5	5.9	12.0	39.6	0.996
Caffeine	61.3	100	-78.7	-81.7	9.2	7.2	0.04	0.15	0.934
Carbamazepine	85.8	89.6	-49.5	-0.24	0.8	5.5	14.0	46.2	0.999
Ciprofloxacin	41.1	66.6	-90.6	-38.7	32	17	0.16	0.51	0.982
Clarithromycin	82.6	94.1	-91.7	-94.2	15	15	54.6	180.2	0.931
Diclofenac	85.9	85.7	-44.1	1.53	2.4	2.3	9.9	32.5	0.996
Dimethomorph	81.8	91.6	-27.3	-40.5	15	16	16.8	55.5	0.988
Erythromycin	69.3	63.4	-98.4	-99.3	5.6	29	68.3	225.2	0.909
Estradiol	87.0	84.9	-73.1	-55.8	15	1.7	9.7	32.0	0.994
Estrone	93.1	81.3	-35.4	367	16	3.3	138	455	0.952
Ibuprofen	84.0	85.0	-53.0	-3.59	6.4	2.8	9.8	32.2	0.997
Methylparaben	NV	NV	NV	NV	NV	NV	NV	NV	NV
Naproxen	80.1	157	156	574	13	19	21.2	70.0	0.989
Progesterone	89.8	83.4	-73.9	-19.3	1.0	5.6	48.0	158.4	0.996
Propylparaben	96.8	100	-14.3	-38.7	17	6.0	3.3	10.9	0.958
Testosterone	86.5	93.7	-76.6	-78.8	4.1	5.0	23.4	77.2	0.985
Tonalide	100	92.6	434	5,570	23	21	58.5	193.1	0.902
Triclocarban	92.7	87.5	-85.1	-7.41	35	4.3	95.5	315.3	0.932
Triclosan	90.1	92.7	54.0	956	2.5	4.1	193.2	637.6	0.988

[1]NV: not validated is used for values that did not meet the validation criteria.

SI-2.3 Risk Assessment

Since bisphenols have been acknowledged as a substance of concern due to their structural similarities to Bisphenol A (BPA), the European Food Safety Authority (EFSA) has been evaluating their potential health risks [9]. The Tolerable Daily Intake (TDI) for BPA was in April 2023 reduced to 0.2 ng/kg body weight per day, reflecting increased concern over its immunotoxic effects [10]. However, EFSA has not established a specific TDI for BPS. Therefore, in the absence of such values for BPS and BPF, the authors selected to apply the current TDI for BPA as a conservative proxy.

Table S5: Consumption rates for tomatoes in Slovenia, acquired from EFSA Food consumption statistics.

Population Group (L2)	Body weight (kg)	Exposure hierarchy (L7)	Number of subjects	Number of consumers	Mean (kg/day)	Standard Deviation	97.5th percentile (kg/day)
Adults - chronic	70	Tomatoes	385	189	0.0297	51.73	0.17742
Adults – acute, consumers only		Tomatoes		240	0,0954	86,96	0,26691
Adolescents - chronic	53	Tomatoes	484	198	0.0158	34.28	0.11646
Adolescents – acute, consumers only		Tomatoes		240	0,0644	65,98	0,23294
Toddlers - chronic	12	Tomatoes	343	156	0.0079	16.45	0.05429
Toddlers – acute, consumers only		Tomatoes		185	0,0294	33,40	0,11671

Table S6: Health-based reference values, including Acceptable Daily Intake (ADI), Acute Reference Dose (ARfD), Tolerable Daily Intake (TDI) or No Observed Adverse Effect Level (NOAEL) for individual CEC.

Compound	µg/kg bw/day	Assessment	Reference
Acetamiprid	5	ADI	[11]
Acetamiprid	5	ARfD	[11]
BPA	0.0002	TDI	[10]
BPS	0.0002	TDI	[10]
Caffeine	5700	ADI	[12]
Carbamazepine	0.16	ADI	[13]
Ciprofloxacin	0.15	ADI	[14]
Dimethomorph	50	ADI	[15]
Dimethomorph	600	ARfD	[15]
Ibuprofen	0.29	ADI	[14]
Progesterone	30	ADI	[16]
Testosterone	2	ADI	[14]
Triclocarban	25000	NOAEL	[17]
Triclosan	8000	NOAEL	[17]

Table S7: Predicted environmental concentrations (PNEC) of each CEC in soil based on PNEC of freshwater.

Compound	$PNEC_{water}$ (µg/L)[18]	Calculated $PNEC_{soil}$ (µg/kg dry weight)
17α-ethynylestradiol	0.000037	0.000006
Acetamiprid	0.037	0.083
Azithromycin	0.019	14.5
Benzophenone	5.4	41.1
beta-estradiol	0.0004	0.0055
BPA	0.24	5.3
BPAF	1.0	123
BPF	5.4	61.5
BPS	12.9	120
Caffeine	1.2	1.4
Carbamazepine	2	19.4
Clarithromycin	0.12	96
Diclofenac	0.04	4.1
Dimethomorph	5.6	24.4
Erythromycin	0.3	234
Estrone	0.0004	2.3
Ibuprofen	0.011	0.028
Naproxen	1.7	16.8
Progesterone	1	9,928
Tonalide	0.023	1.2
Triclocarban	0.011	0.07
Triclosan	0.84	1

SI-3 Results
Results are discussed in terms of measured concentrations and statistical differences. For dry samples, all concentrations are provided based on dry weight (d.w.).

Table S8: CEC concentration (ng/g) in dried sludge used in the experiments.

Compound	Concentration ± sd
17α-ethynylestradiol	< LOQ
Acetamiprid	< LOQ
Azithromycin	58.4 ± 0.8
beta-estradiol	< LOQ
BPA	26,480 ± 4,040
BPAF	23.1 ± 0.9
BPF	206 ± 28
BPS	69 ± 2
Benzophenone	745 ± 70
Caffeine	14.3± 0.2
Carbamazepine	54.6 ± 2.2
Ciprofloxacin	394 ± 47
Clarithromycin	< LOQ
Diclofenac	< LOQ
Dimethomorph	< LOD
Erythromycin	< LOQ
Estrone	< LOD
Ibuprofen	< LOQ
Naproxen	< LOQ
Progesterone	227 ± 29
Propylparaben	< LOD
Testosterone	< LOQ
Tonalide	10,350 ± 2,380
Triclocarban	< LOD
Triclosan	958 ± 42

Table S9: Concentration of CEC in peat substrate at the beginning of the experiment, expressed in ng/g d.w.

	PC		P1		P2		SP1		SP2		SP3		SP4		SP5	
	Mean ± sd	Max	Mean ± sd	Max	Mean ± sd	Max	Mean ± sd	Max	Mean ± sd	Max	Mean ± sd	Max	Mean ± sd	Max	Mean ± sd	Max
17α-ethynylestradiol	<LOQ	<LOQ	330 ± 23	348	3,670 ± 690	4,660	n.d.	n.d.	13.5 ± 28.1	63.8	377 ± 236	784	3,750 ± 650	4,470	n.d.	n.d.
Acetamiprid	n.d.	n.d.	349 ± 37	396	3,490 ± 380	3,970	n.d.	n.d.	14.8 ± 24.8	58.9	352 ± 118	545	2,510 ± 538	3,190	n.d.	n.d.
Azithromycin	<LOQ	<LOQ	122 ± 43	180	1,430 ± 250	1,760	n.d.	n.d.	<LOQ	<LOQ	112 ± 61.3	204	731 ± 185	928	n.d.	n.d.
beta-estradiol	n.d.	n.d.	216 ± 39	251	2,770 ± 610	3,520	n.d.	n.d.	15.4 ± 25.8	61.4	228 ± 202	589	2,440 ± 94.5	2,600	7.5 ± 7.9	13.2
BPA	n.d.	n.d.	369 ± 36	408	4,220 ± 460	4,750	190 ± 38	231	1,330 ± 86.8	1,420	546 ± 249	958	4,210 ± 655	5,060	1,240 ± 560	1,635
BPAF	n.d.	n.d.	526 ± 90	655	2,410 ± 140	2,540	n.d.	n.d.	10.0 ± 21.7	49	446 ± 187	748	1,800 ± 260	2,190	n.d.	n.d.
BPF	<LOQ	<LOQ	336 ± 36	387	3,840 ± 470	4,330	n.d.	n.d.	16.5 ± 32.5	75	370 ± 173	673	2,800 ± 480	3,370	n.d.	n.d.
BPS	2.61 ± 3.17	7.48	362 ± 32	407	2,920 ± 250	3,230	<LOQ	<LOQ	20.6 ± 23.2	62	377 ± 183	436	2,270 ± 330	2,630	9.6 ± 5.7	13.6
Benzophenone	n.d.	n.d.	421 ± 77	538	2,580 ± 180	2,790	<LOQ	<LOQ	202 ± 60	286	432 ± 83.9	503	2,440 ± 230	2,720	103 ± 17	115
Caffeine	n.d.	n.d.	250 ± 62	323	2,530 ± 198	2,840	n.d.	n.d.	8.47 ± 13.7	33	252 ± 54.8	332	1,550 ± 474	2,170	n.d.	n.d.
Carbamazepine	n.d.	n.d.	374 ± 37	414	1,610 ± 75	1,730	n.d.	n.d.	11.2 ± 19.5	45	372 ± 106	530	1,330 ± 170	1,538	n.d.	n.d.
Clarithromycin	<LOQ	<LOQ	134 ± 48	200	1,530 ± 280	1,890	n.d.	n.d.	<LOQ	<LOQ	114 ± 58.3	199	770 ± 190	980	n.d.	n.d.
Diclofenac	<LOQ	<LOQ	156 ± 17	180	1,830 ± 310	2,300	<LOQ	<LOQ	29.5 ± 23.7	64	156 ± 47.7	230	1,320 ± 270	1,640	8.6 ± 2.8	10.6

Dimethomorph	<LOQ	<LOQ	466 ± 94	619	4,780 ± 1,060	6,160	n.d.	n.d.	13.6 ± 19.3	47.9	516 ± 314	1060	4,860 ± 408	5,165	n.d.	n.d.
Erythromycin	<LOQ	<LOQ	93 ± 42	142	2,130 ± 550	2,730	n.d.	n.d.	<LOQ	<LOQ	207 ± 35.5	242	1,577 ± 773	2,603	n.d.	n.d.
Estrone	n.d.	n.d.	413 ± 27	429	3710 ± 490	4,430	9.1 ± 3.3	13	149 ± 35	204	496 ± 237	850	3,965 ± 871	4,984	292 ± 248	467
Ibuprofen	<LOQ	<LOQ	170 ± 58	241	3,310 ± 530	4,070	<LOQ	<LOQ	88 ± 23	121	240 ± 131	472	2,233 ± 223	2,539	60.1 ± 11.7	68.3
Methylparaben	n.d.	n.d.	n.d.	n.d.	9.5 ± 13.4	31	n.d.	n.d.	n.d.	n.d.	n.d.	n.d.	10.35 ± 9.27	20.33	n.d.	n.d.
Naproxen	0.27 ± 0.39	0.94	50.7 ± 6.3	60.9	565 ± 94.1	695	n.d.	n.d.	2.7 ± 4	9.9	52.0 ± 17.5	79.2	455.8 ± 89.4	572.8	0.63 ± 0.30	0.84
Progesterone	<LOQ	<LOQ	327 ± 54	396	3,400 ± 790	4,620	<LOQ	<LOQ	62 ± 37	124	285 ± 185	613	2,551 ± 227	2,523	52.6 ± 45.2	84.5
Propylparaben	n.d.	n.d.	n.d.	n.d.	14.4 ± 16.7	38	n.d.	n.d.	n.d.	n.d.	n.d.	n.d.	24.76 ± 9.41	36.48	n.d.	n.d.
Testosterone	n.d.	n.d.	239 ± 48	285	2140 ± 280	2,520	n.d.	n.d.	10.1 ± 16.7	40	158 ± 140	406	1,217 ± 112	1,326	n.d.	n.d.
Tonalide	n.d.	n.d.	187 ± 234	594	2,850 ± 630	3460	<LOQ	<LOQ	351 ± 166	620	232 ± 92.7	309	3,637 ± 1148	4,963	573 ± 22	589
Triclocarban	<LOQ	<LOQ	574 ± 494	1,450	5,210 ± 1,030	6,370	<LOQ	<LOQ	8.5 ± 9.8	26	352 ± 136	575	1,251 ± 246	1,564	15.1 ± 6.3	19.6
Triclosan	<LOQ	<LOQ	336 ± 37	384	3,640 ± 490	4,290	14.1 ± 3.6	18.4	137 ± 26	168	398 ± 175	667	3,355 ± 707	3,899	138 ± 70.7	188

[1] A higher standard deviation compared to mean value indicates that the compound was quantified in less than two replicates. n.d.: not detected

Table S10: Concentration of CEC in peat substrate at the end of the experiment, expressed in ng/g d.w.

	PC		P1		P2		SP1		SP2		SP3		SP4		SP5	
	Mean ± sd	Max	Mean ± sd	Max	Mean ± sd	Max	Mean ± sd	Max	Mean ± sd	Max	Mean ± sd	Max	Mean ± sd	Max	Mean ± sd	Max
17α-ethynylestradiol	n.d.	n.d.	8.2 ± 8.5	21	72.9 ± 48.1	157	<LOQ	<LOQ	<LOQ	<LOQ	<LOQ	<LOQ	151 ± 145	339	n.d.	n.d.
Acetamiprid	n.d.	n.d.	<LOQ	<LOQ	<LOQ	<LOQ	n.d.	n.d.	n.d.	n.d.	n.d.	n.d.	21.3 ± 22.1	46.1	n.d.	n.d.
Azithromycin	n.d.	n.d.	17.3 ± 16.9	46.2	328 ± 134	445	n.d.	n.d.	n.d.	n.d.	26.9 ± 3.6	31.6	286 ± 80	360	n.d.	n.d.
beta-estradiol	n.d.	n.d.	4.5 ± 3.8	11.0	7.9 ± 2.9	12.8	n.d.	n.d.	n.d.	n.d.	n.d.	n.d.	6.8 ± 2.4	9.45	n.d.	n.d.
BPA	n.d.	n.d.	<LOQ	<LOQ	<LOQ	<LOQ	n.d.	n.d.	n.d.	n.d.	n.d.	n.d.	67 ± 59	132	99 ± 64	173
BPAF	n.d.	n.d.	111 ± 120	256	781 ± 394	445	n.d.	n.d.	n.d.	n.d.	87.9 ± 58.7	169	1,190 ± 247	1,460	n.d.	n.d.
BPF	n.d.	n.d.	5.4 ± 6.7	16.4	n.d.	n.d.	<LOQ	<LOQ	n.d.	n.d.	n.d.	n.d.	7.3 ± 4.7	13.9	<LOQ	<LOQ
BPS	n.d.	n.d.	6.3 ± 9.1	21.3	n.d.	n.d.	n.d.	n.d.	n.d.	n.d.	n.d.	n.d.	2.2 ± 1.5	4.5	n.d.	n.d.
Benzophenone	110 ± 112	303	49.1 ± 47.9	125	59.7 ± 39.8	115	61.4 ± 60.7	139	25.4 ± 26.8	70.2	74.8 ± 60.4	180	110 ± 60.1	191	104 ± 44.5	155
Caffeine	n.d.	n.d.	16.4 ± 16.7	45	13.4 ± 19.1	44	n.d.	n.d.	n.d.	n.d.	<LOQ	<LOQ	63.4 ± 59.6	149	n.d.	n.d.
Carbamazepine	n.d.	n.d.	122 ± 72	202	1050 ± 191	1260	n.d.	n.d.	n.d.	n.d.	221 ± 78	353	941 ± 40	998	n.d.	n.d.
Clarithromycin	n.d.	n.d.	19.2 ± 17.8	49.2	351 ± 145	476	n.d.	n.d.	n.d.	n.d.	27.8 ± 5.2	35.1	314 ± 90.3	401	n.d.	n.d.
Diclofenac	n.d.	n.d.	6.2 ± 7.5	18.6	4.8 ± 4.9	12.2	n.d.	n.d.	<LOQ	<LOQ	<LOQ	<LOQ	43.9 ± 53.3	103	n.d.	n.d.
Dimethomorph	n.d.	n.d.	76.2 ± 86.2	224	622 ± 435	1230	n.d.	n.d.	n.d.	n.d.	36.2 ± 15.4	54.6	466 ± 336	989	n.d.	n.d.
Erythromycin	n.d.	n.d.	17.8 ± 10.7	35.7	206 ± 122	398	n.d.	n.d.	n.d.	n.d.	27.7 ± 7.9	41.8	144 ± 63.4	203	n.d.	n.d.

Estrone	n.d.	n.d.	64.5 ± 136	308	9.8 ± 6.3	20.6	n.d.	n.d.	n.d.	n.d.	n.d.	n.d.	34.0 ± 22.8	70.6	8.54 ± 13.4	23.9
Ibuprofen	n.d.	n.d.	16.8 ± 30.4	70.8	n.d.	n.d.	n.d.	n.d.	n.d.	n.d.	n.d.	n.d.	10.3 ± 6.3	20.2	6.7 ± 9.5	17.7
Methylparaben	n.d.	n.d.	n.d.	n.d.	n.d.	n.d.	n.d.	n.d.	n.d.	n.d.	n.d.	n.d.	n.d.	n.d.	n.d.	n.d.
Naproxen	n.d.	n.d.	n.d.	n.d.	n.d.	n.d.	0.06 ± 0.07	0.19	n.d.	n.d.	n.d.	n.d.	0.18 ± 0.35	0.80	<LOQ	<LOQ
Progesterone	n.d.	n.d.	22.8 ± 35.7	86.3	20.5 ± 6.5	28.9	n.d.	n.d.	n.d.	n.d.	n.d.	n.d.	65.7 ± 20.8	87.4	n.d.	n.d.
Propylparaben	n.d.	n.d.	n.d.	n.d.	n.d.	n.d.	n.d.	n.d.	n.d.	n.d.	n.d.	n.d.	n.d.	n.d.	n.d.	n.d.
Testosterone	n.d.	n.d.	<LOQ	<LOQ	<LOQ	<LOQ	n.d.	n.d.	n.d.	n.d.	n.d.	n.d.	<LOQ	<LOQ	n.d.	n.d.
Tonalide	n.d.	n.d.	216 ± 317	768	1,240 ± 426	1,830	n.d.	n.d.	81.8 ± 49.6	121	32.2 ± 47.2	115	1,250 ± 284	1,740	353 ± 100	463
Triclocarban	8.1 ± 12.2	29.7	182 ± 134	373	3,640 ± 1,240	5,022	n.d.	n.d.	13.5 ± 5.2	21.9	251 ± 114	410	3,605 ± 568	4,196	59.4 ± 23.4	82.3
Triclosan	n.d.	n.d.	114 ± 92.6	219	563 ± 426	1,233	10.1 ± 10.3	25.6	20.1 ± 23.9	57.5	73.4 ± 69.7	182	1,390 ± 1,050	2,540	131 ± 34.6	164

[1] A higher standard deviation compared to mean value indicates that the compound was quantified in less than two replicates. n.d.: not detected

Table S11: Analysis of substrate samples at the end of the experiment.

	pH in $CaCl_2$	C_{total}	N_{total}	C_{total} / N_{total}	NH_4-N	NO_3-N	EC
		[%]	[%]		[mg/100g]		[µS/cm]
P	6.0	37.5	0.9	39.7	11.7	22.4	1,756.3
P1	5.9	39.2	1.0	40.2	12.7	31.0	1,965.2
P2	5.9	39.0	0.9	41.7	8.9	25.9	2,089.3
SP1	6.1	38.2	1.0	37.3	15.9	33.6	1,866.6
SP2	6.4	33.3	1.6	20.8	18.2	6.4	1,735.3
SP3	6.0	39.1	1.0	38.3	18.4	24.6	1,838.0
SP4	6.4	34.3	1.7	21.1	21.1	10.4	1,792.3
SP5	6.4	35.5	1.3	27.9	16.6	8.3	2,271.3

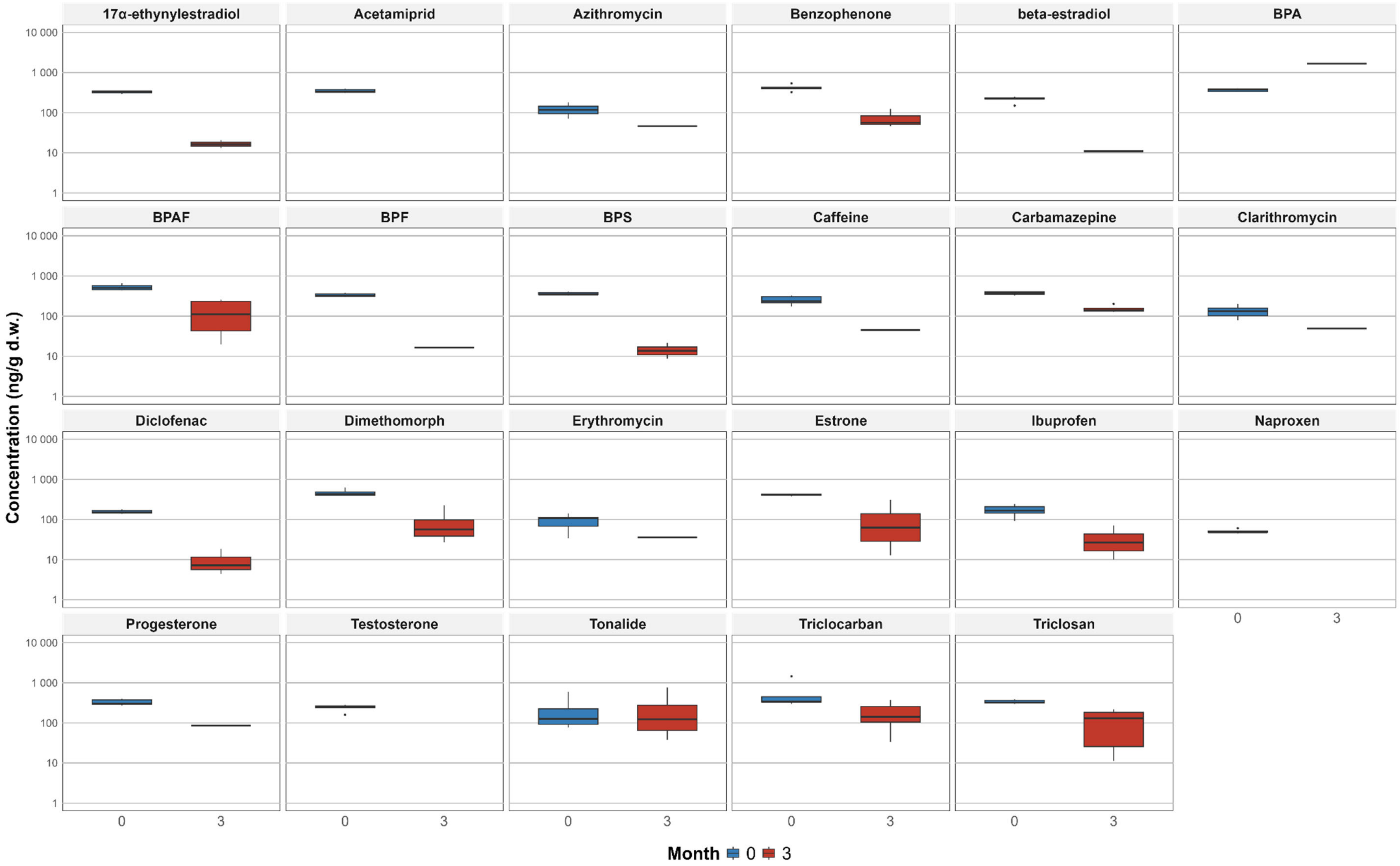

Fig S5: Boxplots showing CEC concentrations at month 0 and month 3 for P1.

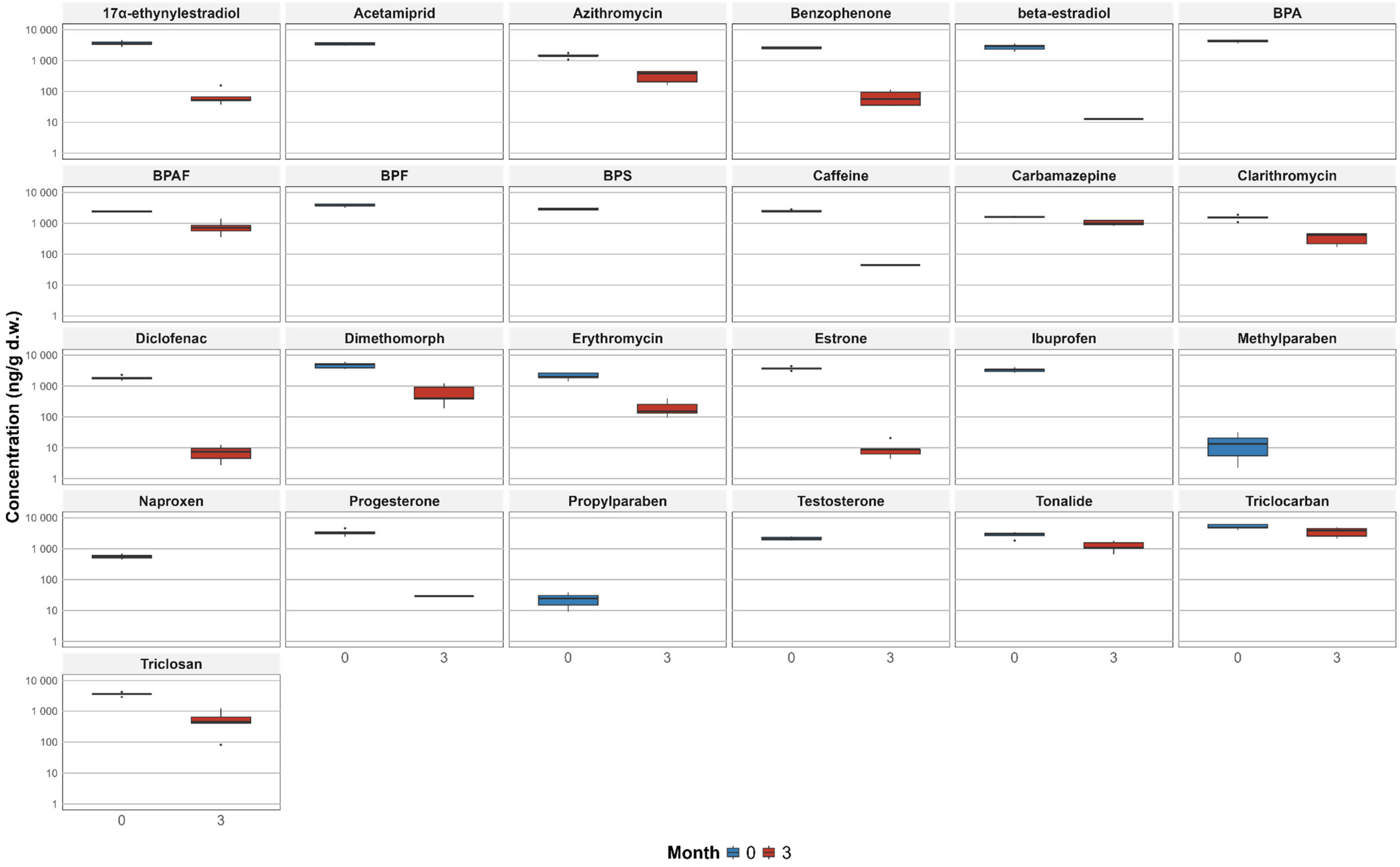

Fig S6: Boxplots showing CEC concentrations at month 0 and month 3 for P2.

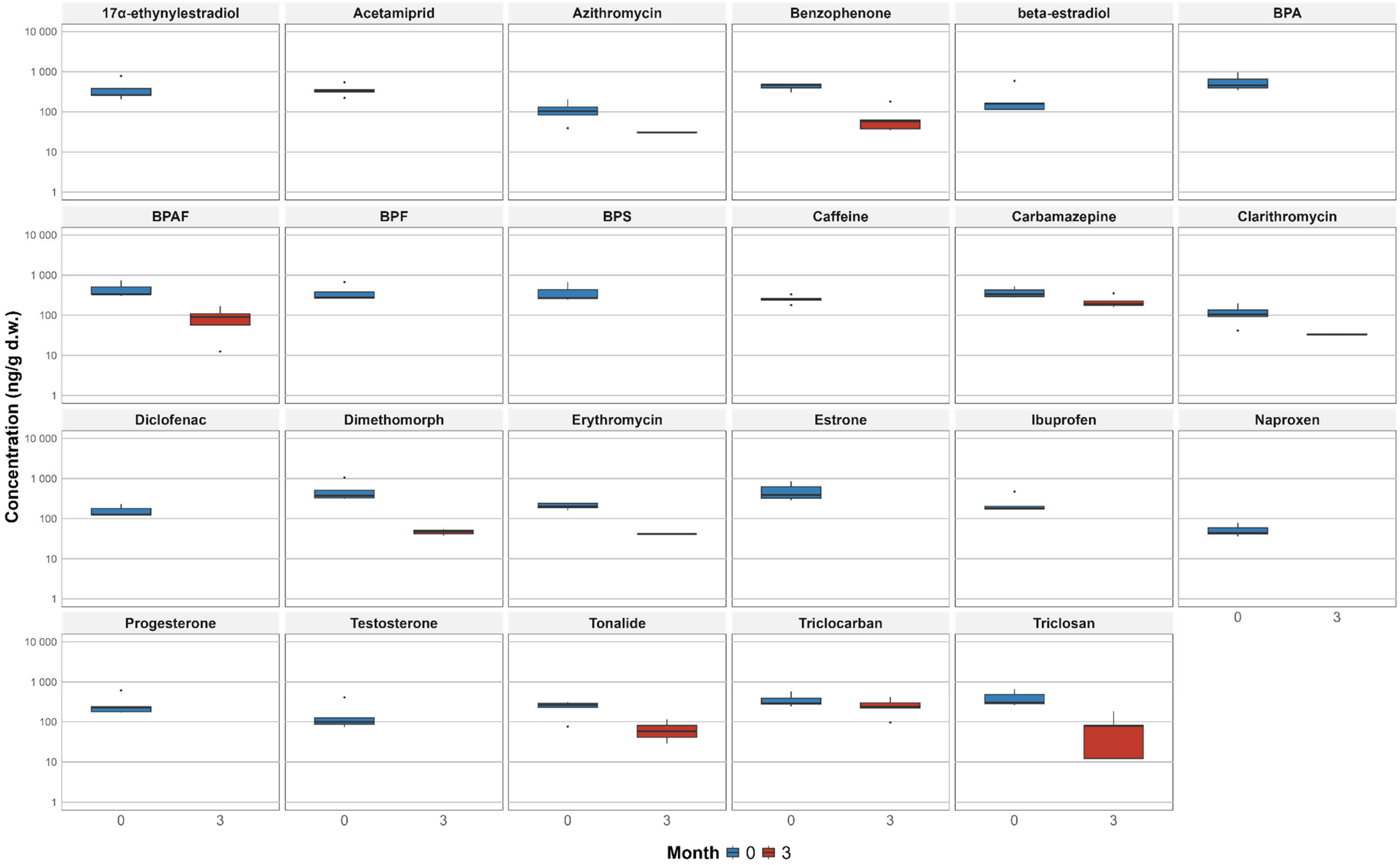

Fig S7: Boxplots showing CEC concentrations at month 0 and month 3 for SP3.

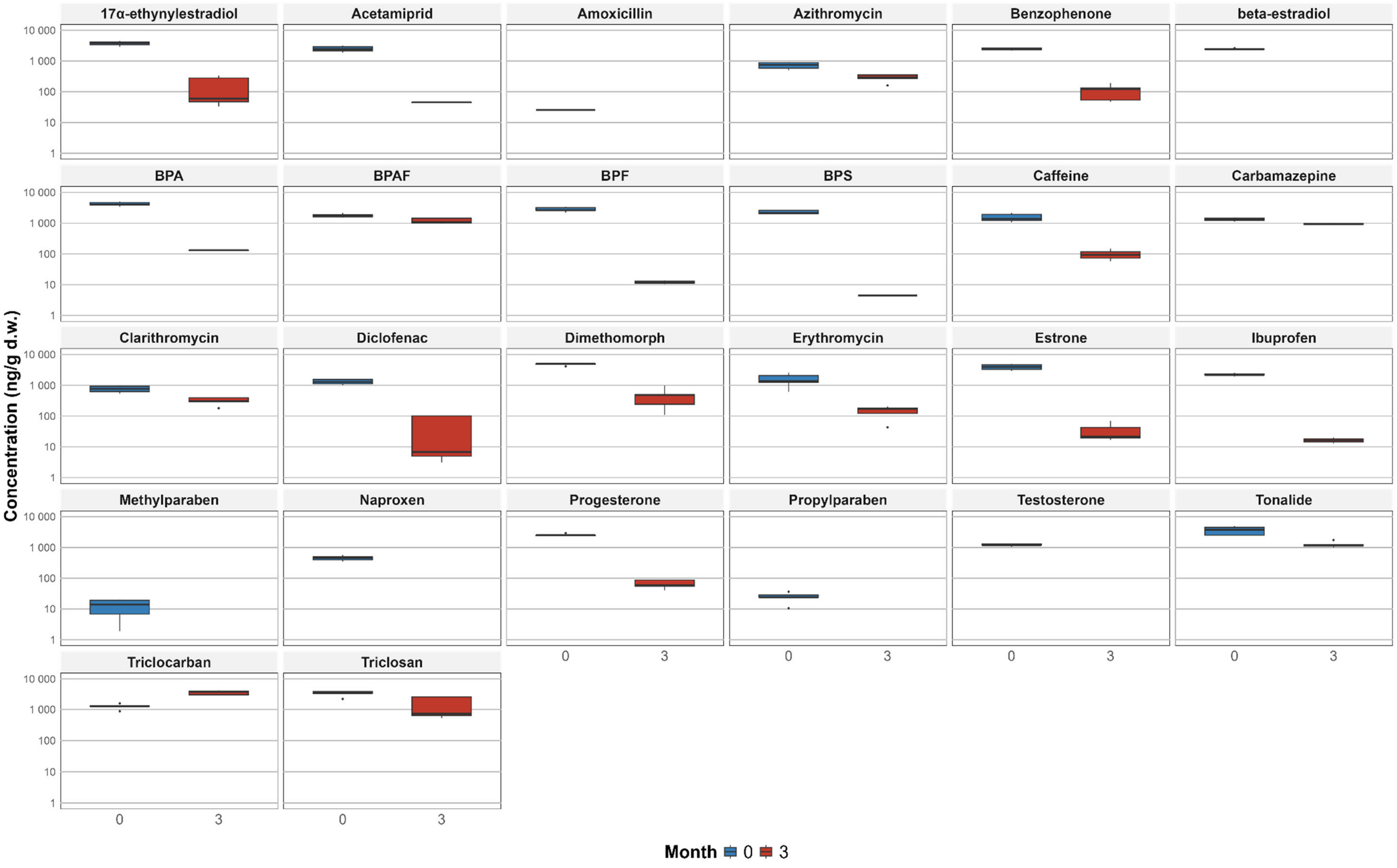

Fig S8: Boxplots showing CEC concentrations at month 0 and month 3 for SP4.

Table S12: Month-0 vs Month-3 comparisons per compound and treatment.

	P1	P2	SP1	SP2	SP3	SP4	SP5
17α-ethynylestradiol	***	***	n.d.	n.s.	*	***	n.d.
Acetamiprid	***	***	n.d.	n.s.	**	***	n.d.
Amoxicillin	n.d.	n.d.	n.d.	n.d.	n.d.	**	n.d.
Azithromycin	**	***	n.d.	n.d.	*	**	n.s.
beta-estradiol	***	***	n.d.	***	*	***	n.s.
BPA	*	***	***	n.s.	**	***	n.d.
BPAF	***	***	n.d.	n.s.	**	**	n.d.
BPF	***	***	n.d.	n.s.	**	***	n.d.
BPS	***	***	n.d.	n.s.	**	***	n.s.
Benzophenone	***	***	n.d.	***	***	***	n.s.
Caffeine	***	***	n.d.	n.s.	***	**	n.d.
Carbamazepine	***	***	n.d.	n.s.	*	**	n.d.
Clarithromycin	**	***	n.d.	n.d.	*	**	n.d.
Diclofenac	***	***	n.d.	*	***	***	n.s.
Dimethomorph	***	***	n.d.	n.s.	*	***	n.d.

Erythromycin	**	***	n.d.	n.d.	***	**	n.d.
Estrone	**	***	**	***	**	***	n.s.
Ibuprofen	***	***	n.d.	***	**	***	*
Methylparaben	n.d.	n.s.	n.d.	n.d.	n.d.	*	n.d.
Naproxen	***	***	n.d.	n.s.	**	***	n.s.
Progesterone	***	***	n.d.	*	*	***	n.s.
Propylparaben	n.d.	n.s.	n.d.	n.d.	n.d.	**	n.d.
Testosterone	***	***	n.d.	n.s.	*	***	n.d.
Tonalide	n.s.	**	n.d.	*	**	**	*
Triclocarban	n.s.	*	n.d.	n.s.	n.s.	n.s.	n.s.
Triclosan	**	***	n.s.	***	***	**	n.s.

n.d. : not determined, n.s. : not significant ($p \geq 0.05$), * = significant at $p < 0.05$, ** = significant at $p < 0.01$, *** = significant at $p < 0.00$

Table S13: Average concentration of CEC in plant parts, expressed in ng/g d.w.

	Roots							
	PC	P1	P2	SP1	SP2	SP3	SP4	SP5
17α-ethynylestradiol	<LOQ	<LOQ	<LOQ	<LOQ	<LOQ	<LOQ	53.5	<LOQ
Acetamiprid	<LOQ	<LOQ	29.0	<LOQ	<LOQ	<LOQ	<LOQ	<LOQ
BPA	<LOQ	<LOQ	<LOQ	<LOQ	<LOQ	<LOQ	47.3	<LOQ
Carbamazepine	<LOQ	<LOQ	90.3	<LOQ	<LOQ	<LOQ	235	<LOQ
Diclofenac	<LOQ	<LOQ	<LOQ	<LOQ	<LOQ	<LOQ	55.5	<LOQ
Ibuprofen	<LOQ	<LOQ	<LOQ	<LOQ	54.4	<LOQ	<LOQ	<LOQ
Triclosan	<LOQ	<LOQ	129	<LOQ	<LOQ	<LOQ	869	<LOQ
	Leaves							
Acetamiprid	<LOQ	<LOQ	<LOQ	<LOQ	<LOQ	<LOQ	740	<LOQ
BPS	<LOQ	30.4	<LOQ	<LOQ	<LOQ	<LOQ	<LOQ	<LOQ
Carbamazepine	<LOQ	<LOQ	301	<LOQ	<LOQ	<LOQ	678	<LOQ
Dimethomorph	<LOQ	<LOQ	<LOQ	<LOQ	<LOQ	<LOQ	168	<LOQ
	Stems							
Carbamazepine	<LOQ	<LOQ	74.6	<LOQ	<LOQ	<LOQ	236	<LOQ

Table S14: Root Concentration Factor (RCF) calculated based on peat substrate measurement.

C roots/ C soil	PC	P1	P2	SP1	SP2	SP3	SP4	SP5
17α-ethynylestradiol	NA	NA	NA	NA	NA	NA	0.025	NA
Acetamiprid	NA	NA	0.013	NA	NA	NA	NA	NA
BPA	NA	NA	NA	NA	NA	NA	0.02	NA
Carbamazepine	NA	NA	0.056	NA	NA	NA	0.18	NA
Diclofenac	NA	NA	NA	NA	NA	NA	0.08	NA
Ibuprofen	NA	NA	NA	NA	0.62	NA	NA	NA
Triclosan	NA	NA	0.035	NA	NA	NA	0.26	NA

[1]NA: Not applicable due to lack of values.

Table S15: Concentration of CEC quantified in tomato samples, including composite samples per plant (1-5) and composite samples per harvest season (A-C).

a.

LOD	8.0	8.2	8.1	87.2	2.6	11.9	5.2	1.1	2.0	4.4	2.6	5.4
LOQ	26.4	26.9	26.8	287.7	8.6	39.2	17.1	3.6	6.8	14.6	8.7	17.8
Sample Name	Acetamiprid	BPA	BPS	Caffeine	Carbamazepine	Ciprofloxacin	Dimethomorph	Ibuprofen	Progesterone	Testosterone	Triclocarban	Triclosan
PC_1_F	-	-	-	-	-	-	-	<LOQ	-	-	-	18.7
PC_5_F	-	-	<LOQ	-	-	-	-	<LOQ	-	-	-	19.0
P1_2_F	-	-	-	-	-	-	-	15.4	-	-	<LOQ	<LOQ
P1_5_F	-	-	<LOQ	-	<LOQ	-	-	36.3	-	-	-	<LOQ
P2_1_F	-	28.1	-	-	<LOQ	-	-	30.5	-	-	<LOQ	<LOQ
P2_3_F	-	-	-	-	<LOQ	-	-	11.3	-	-	-	<LOQ
P2_A_F	<LOQ	-	-	-	11.3	-	-	<LOQ	-	-	-	-
P2_C_F	-	<LOQ	-	-	<LOQ	-	-	-	-	-	<LOQ	-
SP1_1_F	-	-	-	-	-	-	-	<LOQ	-	-	-	<LOQ
SP1_2_NF	-	-	-	-	-	-	-	<LOQ	-	-	-	17.9
SP1_5_NF	-	-	-	-	<LOQ	-	-	11.5	-	-	<LOQ	-
SP1_B_NF	-	-	-	<LOQ	-	-	-	20.0	-	-	-	-
SP2_2_NF	<LOQ	-	<LOQ	-	<LOQ	-	-	7.5	-	-	-	<LOQ
SP2_3_F	-	-	-	-	-	241	-	-	-	-	-	<LOQ
SP2_5_NF	-	-	-	-	-	-	-	4.5	-	-	-	<LOQ

SP2_B_F	-	-	-	381	<LOQ	372	-	4.5	<LOQ	-	<LOQ	-
SP2_B_NF	<LOQ	-	<LOQ	<LOQ	18.1	-	-	4.4	-	-	-	-
SP3_1_F	-	-	-	-	<LOQ	-	-	3.8	-	-	-	-
SP3_A_NF	-	-	-	<LOQ	<LOQ	-	-	-	-	-	-	-
SP3_B_F	-	-	<LOQ	<LOQ	10.2	-	-	-	-	23.2	-	-
SP3_B_NF	-	-	-	<LOQ	<LOQ	-	-	<LOQ	-	-	-	-
SP3_C_NF	-	-	-	<LOQ	<LOQ	-	-	53.1	-	<LOQ	-	-
SP4_1_F	-	-	-	-	<LOQ	-	-	<LOQ	-	-	-	-
SP4_2_NF	<LOQ	-	-	<LOQ	20.0	229	-	<LOQ	-	-	-	-
SP4_3_F	<LOQ	-	-	-	15.6	176	56.5	<LOQ	-	-	-	-
SP4_5_NF	-	-	-	-	10.2	-	-	<LOQ	-	-	-	-
SP4_A_NF	47.1	-	-	<LOQ	42.7	-	-	-	-	-	-	-
SP4_B_F	<LOQ	-	-	<LOQ	32.3	408	84.1	<LOQ	-	-	-	-
SP4_B_NF	<LOQ	-	-	<LOQ	28.6	396	-	<LOQ	-	-	-	-
SP4_C_F	-	-	-	<LOQ	18.3	-	-	<LOQ	-	-	-	-
SP5_1_F	-	-	241	-	<LOQ	-	-	108	-	-	-	<LOQ
SP5_2_NF	-	-	-	-	-	-	-	12.1	-	-	-	<LOQ
SP5_3_NF	-	-	<LOQ	-	19.8	-	-	12.5	7.01	-	11.0	33.1
SP5_B_F	-	-	-	<LOQ	40.1	-	-	9.0	<LOQ	-	<LOQ	-

SP5_C_F	-	<LOQ	172	<LOQ	-	-	-	<LOQ	-	-	-	-
SP5_C_NF	-	<LOQ	-	<LOQ	-	-	-	<LOQ	-	-	-	-

[1] - =Not detected

Table S16: Bioconcentration Factor (BCF) calculated based on peat substrate concentration.

C tomato/ C soil	PC	P1	P2	SP1	SP2	SP3	SP4	SP5
Acetamiprid	NA	NA	NA	NA	NA	NA	0.02	NA
BPA	NA	NA	0.006	NA	NA	NA	NA	NA
BPS	NA	NA	NA	NA	NA	NA	NA	21
Caffeine	NA	NA	NA	NA	35	NA	NA	NA
Carbamazepine	NA	NA	0.01	NA	1.6	0.03	0.02	12
Dimethomorph	NA	NA	NA	NA	NA	NA	0.01	NA
Ibuprofen	NA	0.1	0.006	6.1	0.06	0.1	NA	0.6
Progesterone	NA	NA	NA	NA	NA	NA	NA	0.1
Testosterone	NA	NA	NA	NA	NA	0.1	NA	NA
Triclocarban	NA	NA	NA	NA	NA	NA	NA	0.7
Triclosan	12	NA	NA	1.3	NA	NA	NA	0.2

[1]NA: Not applicable due to lack of values.

Table S17: Tomato yield production from different treatments and seasons.

Treatment	Season	Fertilising	Replicate	Amount	Weight
PC	1	F	5	6	1,299
PC	2	F	5	31	4,897
PC	3	F	5	13	2,119
P1	1	F	5	2	352
P1	2	F	5	27	3,640
P1	3	F	5	12	2,065
P2	1	F	5	8	1,341
P2	2	F	5	41	5,235
P2	3	F	5	25	3,346
SP1	1	F	2	1	115
SP1	2	F	2	4	530
SP1	3	F	2	9	1,518
SP1	1	NF	3	2	502
SP1	2	NF	3	12	1,122
SP1	3	NF	3	0	0
SP2	1	F	2	1	187
SP2	2	F	2	9	1,405
SP2	3	F	2	8	1,032
SP2	1	NF	3	2	448
SP2	2	NF	3	22	3,097
SP2	3	NF	3	0	0
SP3	1	F	2	1	322
SP3	2	F	2	4	420
SP3	3	F	2	6	1,017
SP3	1	NF	3	2	470
SP3	2	NF	3	14	1,911
SP3	3	NF	3	3	149

SP4	1	F	2	1	196
SP4	2	F	2	18	2,219
SP4	3	F	2	5	819
SP4	1	NF	3	1	285
SP4	2	NF	3	13	1,747
SP4	3	NF	3	0	0
SP5	1	F	1	2	301
SP5	2	F	1	6	642
SP5	3	F	1	3	471
SP5	1	NF	2	2	382
SP5	2	NF	2	4	307
SP5	3	NF	2	0	0

* F: fertilised, NF: non-fertilised.

Table S18: Estimated Daily Intake (EDI) as mg/kg per body weight per day.

	PC	
	Triclosan	
Slovenia	Average	High
Adults	4.0E-07	2.4E-06
Adolescents	2.8E-07	2.1E-06
Toddlers	1.7E-07	1.5E-06

	P1	
	Ibuprofen	
Slovenia	Average	High
Adults	7.7E-07	4.6E-06
Adolescents	5.4E-07	4.0E-06
Toddlers	3.3E-07	2.9E-06

	P2					
	BPA		Carbamazepine		Ibuprofen	
Slovenia	Average	High	Average	High	Average	High
Adults	**6.0E-07**	**3.6E-06**	2.4E-07	1.4E-06	6.5E-07	3.9E-06
Adolescents	**4.2E-07**	**3.1E-06**	1.7E-07	1.2E-06	4.6E-07	3.3E-06
Toddlers	**2.6E-07**	**2.3E-06**	1.0E-07	9.1E-07	2.8E-07	2.5E-06

	SP1			
	Ibuprofen		Triclosan	
Slovenia	Average	High	Average	High
Adults	4.2E-07	2.5E-06	3.8E-07	2.3E-06
Adolescents	3.0E-07	2.2E-06	2.7E-07	2.0E-06
Toddlers	1.8E-07	1.6E-06	1.6E-07	1.5E-06

	SP2							
	Caffeine		Carbamazepine		Ciprofloxacin		Ibuprofen	
Slovenia	Average	High	Average	High	Average	High	Average	High

Adults	8.1E-06	4.8E-05	3.9E-07	2.3E-06	7.9E-06	4.7E-05	1.6E-07	9.6E-07
Adolescents	5.7E-06	4.2E-05	2.7E-07	2.0E-06	5.6E-06	4.1E-05	1.1E-07	8.3E-07
Toddlers	3.5E-06	3.1E-05	1.7E-07	1.5E-06	3.4E-06	3.0E-05	6.9E-08	6.1E-07

	SP3					
	Carbamazepine		Ibuprofen		Testosterone	
Slovenia	Average	High	Average	High	Average	High
Adults	2.2E-07	1.3E-06	1.1E-06	6.7E-06	4.9E-07	2.9E-06
Adolescents	1.5E-07	1.1E-06	7.9E-07	5.8E-06	2.1E-07	1.9E-06
Toddlers	9.4E-08	8.3E-07	4.9E-07	4.3E-06	3.5E-07	2.5E-06

	SP4							
	Acetamiprid		Carbamazepine		Ciprofloxacin		Dimethomorph	
Slovenia	Average	High	Average	High	Average	High	Average	High
Adults	1.0E-06	6.0E-06	9.1E-07	5.4E-06	8.7E-06	5.2E-05	1.8E-06	1.1E-05
Adolescents	7.0E-07	5.2E-06	6.4E-07	4.7E-06	6.1E-06	4.5E-05	7.7E-07	6.8E-06
Toddlers	4.3E-07	3.8E-06	3.9E-07	3.5E-06	3.7E-06	3.3E-05	1.3E-06	9.2E-06
Slovenia	Acute exposure (IESTI model)						Acute exposure (IESTI model)	
Adults	1.8E-04						3.2E-04	
Adolescents	2.1E-04						6.1E-04	
Toddlers	4.6E-04						1,4E-03	

	SP5											
	BPS		Carbamazepine		Ibuprofen		Progesterone		Triclosan		Triclocarban	
Slovenia	Average	High	Average	High	Average	High	Average	High	Average	High	Average	High
Adults	**5.1E-06**	**3.1E-05**	8.5E-07	5.1E-06	2.3E-06	1.4E-05	1.5E-07	8.9E-07	7.0E-07	4.2E-06	2.3E-07	1.4E-06
Adolescents	**3.6E-06**	**2.7E-05**	6.0E-07	4.4E-06	1.6E-06	1.2E-05	1.0E-07	7.7E-07	4.9E-07	3.6E-06	1.6E-07	1.2E-06
Toddlers	**2.2E-06**	**2.0E-05**	3.7E-07	3.2E-06	9.9E-07	8.7E-06	6.4E-08	5.7E-07	3.0E-07	2.7E-06	1.0E-07	8.9E-07

[1]Dietary exposure exceeding the corresponding Acceptable Daily Intake are marked with bold.

Table S19: Hazard Quotients for CECs in tomato fruits.

	PC	
	Triclosan	
	Average	High
Adults	5E-08	3E-07
Adolescents	4E-08	3E-07
Toddlers	2E-08	2E-07

	P1	
	Ibuprofen	
Slovenia	Average	High
Adults	0.003	0.02
Adolescents	0.002	0.01
Toddlers	0.001	0.01

	P2					
	BPA		Carbamazepine		Ibuprofen	
Slovenia	Average	High	Average	High	Average	High
Adults	**3.0**	**18**	0.001	0.009	0.002	0.01
Adolescents	**2.1**	**15**	0.001	0.008	0.002	0.01
Toddlers	**1.3**	**11**	0.001	0.006	0.001	0.009

	SP1			
	Ibuprofen		Triclosan	
Slovenia	Average	High	Average	High
Adults	0.0015	0.009	5E-08	3E-07
Adolescents	0.001	0.008	3E-08	2E-07
Toddlers	0.0006	0.006	2E-08	2E-07

	SP2							
	Caffeine		Carbamazepine		Ciprofloxacin		Ibuprofen	
Slovenia	Average	High	Average	High	Average	High	Average	High

Adults	1E-06	8E-06	0.002	0.01	0.05	0.3	0.0006	0.003
Adolescents	1E-06	7E-06	0.002	0.01	0.04	0.3	0.0004	0.003
Toddlers	6E-07	5E-06	0.001	0.01	0.02	0.2	0.0002	0.002

	SP3					
	Carbamazepine		Ibuprofen		Testosterone	
Slovenia	Average	High	Average	High	Average	High
Adults	0.001	0.008	0.004	0.02	0.0002	0.001
Adolescents	0.001	0.007	0.003	0.02	0.0001	0.0009
Toddlers	0.001	0.005	0.002	0.01	0.0002	0.001

	SP4							
	Acetamiprid		Carbamazepine		Ciprofloxacin		Dimethomorph	
Slovenia	Average	High	Average	High	Average	High	Average	High
Adults	0.0002	0.001	0.006	0.03	0.05	0.3	4E-05	2E-04
Adolescents	0.0001	0.001	0.004	0.03	0.04	0.3	2E-05	1E-04
Toddlers	0.0001	0.0008	0.002	0.02	0.02	0.2	3E-05	2E-04
Slovenia	Acute exposure						Acute exposure	
Adults	0.04						0.0005	
Adolescents	0.04						0.0006	
Toddlers	0.09						0.001	

	SP5											
	BPS		Carbamazepine		Ibuprofen		Progesterone		Triclosan		Triclocarban	
Slovenia	Average	High	Average	High	Average	High	Average	High	Average	High	Average	High
Adults	**26**	**153**	0.01	0.03	0.01	0.05	5E-06	3E-05	9E-08	5E-07	9E-09	6E-08
Adolescents	**18**	**133**	0.004	0.03	0.01	0.04	3E-06	3E-05	6E-08	5E-07	7E-09	5E-08
Toddlers	**11**	**98**	0.002	0.02	0.00	0.03	2E-06	2E-05	4E-08	3E-07	4E-09	4E-08

Table S20: Ecological risk assessment results based on Risk Quotient (RQ).

	PC	P1	P2	SP1	SP2	SP3	SP4	SP5
17α-ethynylestradiol	NA	1.4E+06	1.2E+07	NA	NA	NA	2.5E+07	NA
Acetamiprid	NA	NA	NA	NA	NA	NA	0.18	NA
Azithromycin	NA	1.2	22.5	NA	NA	1.8	19.7	NA
beta-estradiol	NA	813	1,435	NA	NA	NA	1,225	NA
BPA	NA	NA	NA	NA	NA	NA	12.7	18.9
BPAF	NA	0.91	6.35	NA	NA	0.7	9.7	NA
BPF	NA	0.09	NA	NA	NA	NA	0.12	NA
BPS	NA	0.05	NA	NA	NA	NA	0.02	NA
Benzophenone	2.7	1.2	1.4	1.5	0.62	1.8	2.7	2.5
Caffeine	NA	11.2	9.2	NA	NA	NA	43.4	NA
Carbamazepine	NA	6.3	54.1	NA	NA	11.4	48.5	NA
Clarithromycin	NA	0.20	3.6	NA	NA	0.3	3.3	NA
Diclofenac	NA	1.5	1.2	NA	NA	NA	10.8	NA
Dimethomorph	NA	3.1	25.5	NA	NA	1.5	19.1	NA
Erythromycin	NA	0.08	0.88	NA	NA	0.12	0.61	NA
Estrone	NA	28.1	4.3	NA	NA	NA	14.8	3.7
Ibuprofen	NA	594	NA	NA	NA	NA	366	238
Naproxen	NA	NA	NA	0.004	NA	NA	0.01	NA
Progesterone	NA	0.002	0.002	NA	NA	NA	0.01	NA
Tonalide	NA	181	1,038	NA	68.5	27	1,046	296
Triclocarban	105	2,350	47,237	NA	175	3,258	46,730	770
Triclosan	NA	114	563	10.1	20	73.4	1390	131

[1]NA: Not applicable due to lack of measured environmental concentration values.

SI-5 References

1. Andreasidou, E.; Kovačič, A.; Manzano-Sánchez, L.; Heath, D.; Kosjek, T.; Pintar, M.; Maršič, N.K.; Blaznik, U.; Fernán-dez-Alba, A.R.; Hernando, M.D.; et al. Uptake of Emerging Contaminants in Tomato Plants: A Field Study on Treated Wastewater Reuse. Environ. Int. 2025, 205, 109916. https://doi.org/10.1016/J.ENVINT.2025.109916.
2. ISO 11464:2006; Soil Quality—Pretreatment of Samples for Physico-Chemical Analysis. International Organization for Standardization: Geneva, Switzerland, 2006.
3. ISO 10694:1995; Soil Quality—Determination of Organic and Total Carbon after Dry Combustion (Elementary Analysis). International Organization for Standardization: Geneva, Switzerland, 1995.
4. ISO 13878:1998; Soil Quality—Determination of Total Nitrogen Content by Dry Combustion ("Elemental Analysis"). Interna-tional Organization for Standardization: Geneva, Switzerland, 1998.
5. Egnér, H.; Riehm, H.; Domingo, W.R. Untersuchungen Uber Die Chemishe Boden-Analyse Als Grundlage Fur Die Beurteilung Des Nahrsoffzustandes Der Boden. II. Chemiche Extraktionsmethoden Zur Phosphor Und Kalimbestimmung. Kungliga Lantbrukshögskolans Annaler; Uppsala, Sweden, 1960; pp. 199–215.
6. ISO 10390:2021; Soil, Treated Biowaste and Sludge—Determination of PH. International Organization for Standardization: Geneva, Switzerland, 2021
7. Wenzl, T.; Haedrich, J.; Schaechtele, A.; Robouch, P.; Stroka, J.; Burdaspal, P.; Kapp, T.; Amlund, H.; Jensen, U.; Patriarca, M.; et al. Guidance Document on the Estimation of LOD and LOQ for Measurements in the Field of Contaminants in Feed and Food; Pub-lications Office of the European Union, Luxembourg, 2016.
8. European Commission. DG-SANTE Guidance Document on the Analytical Quality Control and Method Validation Procedures for Pesticide Residues in Food and Feed. No SANTE 11312/2021 V2; European Commission: Brussels, Belgium, 2021.
9. FitzGerald, R.; Van Loveren, H.; Civitella, C.; Castoldi, A.F.; Bernasconi, G. Assessment of New Information on Bisphenol S (BPS) Submitted in Response to the Decision 1 under REACH Regulation (EC) No 1907/2006. EFSA Supporting Publication: Parma, Italy, 2020, 17, 1844E. https://doi.org/10.2903/sp.efsa.2020.EN-1844.
10. Lambré, C.; Barat Baviera, J.M.; Bolognesi, C.; Chesson, A.; Cocconcelli, P.S.; Crebelli, R.; Gott, D.M.; Grob, K.; Lampi, E.; Mengelers, M.; et al. Re-Evaluation of the Risks to Public Health Related to the Presence of Bisphenol A (BPA) in Foodstuffs. EFSA J. 2023, 21, e06857. https://doi.org/10.2903/J.EFSA.2023.6857.
11. Hernandez-Jerez, A.; Coja, T.; Paparella, M.; Price, A.; Henri, J.; Focks, A.; Louisse, J.; Terron, A.; Binaglia, M.; Guajardo, I.M.; et al. Statement on the Toxicological Properties and Maximum Residue Levels of Acetamiprid and Its Metabolites. EFSA J. 2024, 22, e8759. https://doi.org/10.2903/j.efsa.2024.8759.
12. EFSA Panel on Dietetic Products, Nutrition and Allergies (NDA). Scientific Opinion on the Safety of Caffeine. EFSA J. 2015, 13, 425. https://doi.org/10.2903/J.EFSA.2015.4102.
13. Scientific Opinion on "Draft Environmental Quality Standards for Priority Substances under the Water Framework Di-rective"—Carbamazepine—European Commission. Available online: https://health.ec.europa.eu/publications/scientific-opinion-draft-environmental-quality-standards-priority-substances-under-water-framework-6_en (accessed on 25 May 2025).
14. Khan, U.; Nicell, J. Human Health Relevance of Pharmaceutically Active Compounds in Drinking Water. AAPS J. 2015, 17, 558–585. https://doi.org/10.1208/s12248-015-9729-5.
15. Alvarez, F.; Arena, M.; Auteri, D.; Binaglia, M.; Castoldi, A.F.; Chiusolo, A.; Colagiorgi, A.; Colas, M.; Crivellente, F.; De Lentdecker, C.; et al. Peer Review of the Pesticide Risk Assessment of the Active Substance Dimethomorph. EFSA J. 2023, 21, e08032. https://doi.org/10.2903/j.efsa.2023.8032.
16. World Health Organization Evaluations of the Joint FAO/WHO Expert Committee on Food Additives (JECFA)—Progesterone. Available online: https://apps.who.int/food-additives-contaminants-jecfa-database/Home/Chemical/3580 (accessed on 11 September 2025).

17. Scientific Committee on Consumer Safety Scientific. Advice on the Safety of Triclocarban and Triclosan as Substances with Potential Endocrine Disrupting Properties in Cosmetic Products; Publications Office of the European Union: Luxembourg, Luxembourg, 2023.
18. NORMAN Ecotoxicology Database Available online: https://www.norman-network.com/nds/ecotox/lowestPnecsIndex.php?checkSelect=1 (accessed on 8 April 2025).
